# 3D-Printed Functionally Graded PCL-HA Scaffolds with Multi-Scale Porosity

**DOI:** 10.1021/acsomega.4c06820

**Published:** 2025-02-14

**Authors:** Hatice Kubra Bilgili, Mehmet Serhat Aydin, Mervenaz Sahin, Sevilay Burcu Sahin, Sibel Cetinel, Gullu Kiziltas

**Affiliations:** † Department of Material Science and Nanoengineering, Faculty of Engineering and Natural Sciences, Sabanci University, Istanbul 34956, Turkey; ‡ Division of Human Mechanical Systems and Design, Graduate School of Engineering, Hokkaido University, Sapporo 060-8628, Japan; § Center for Translational Oral Research (TOR), Department of Clinical Dentistry, Faculty of Medicine, University of Bergen, Bergen 5009, Norway; ∥ Department of Molecular Biology, Genetics and Bioengineering, Faculty of Engineering and Natural Sciences, Sabanci University, Istanbul 34956, Turkey; ⊥ Sabanci University Nanotechnology Research and Application Center, Istanbul 34956, Turkey; # Department of Mechatronics, Faculty of Engineering and Natural Sciences, Sabanci University, Istanbul 34956, Turkey

## Abstract

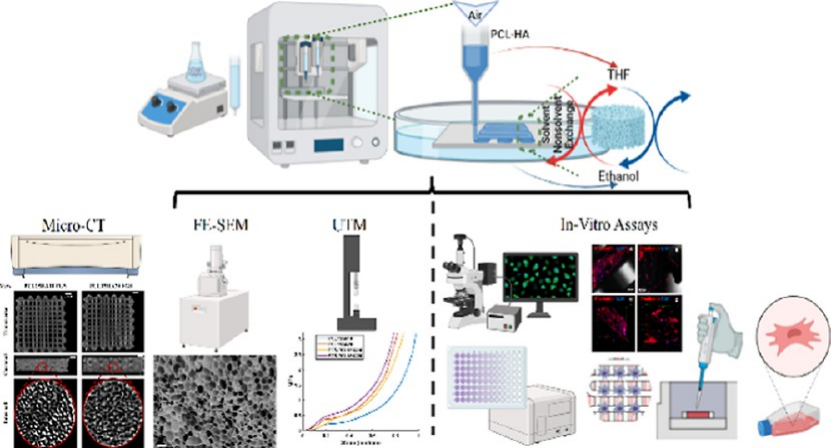

Functionally graded scaffolds (FGSs) designed for bone tissue regeneration exhibit three-dimensional (3D) constructs with spatially varying pores, mirroring the natural bone structure, aiming to offer temporary support and a conducive environment for cells during tissue regeneration in defect sites. While existing research on FGSs has primarily focused on altering pore architecture and tuning biomechanical properties for improved tissue regeneration, limited exploration exists on 3D spatially varying FGSs with multiscale porosity to closely mimic natural bone. In this study, we fabricated and investigated FGSs with macropores varying radially and longitudinally, along with micropores within the struts. Utilizing nonsolvent-induced phase separation integrated with 3D printing, we printed poly(ε-caprolactone) (PCL)/hydroxyapatite (HA) composite scaffolds with both uniform and FG geometries. Two HA content variations (10 and 20 wt %) were employed to assess their impact on scaffold properties. Rheological analysis of polymer suspensions gauged the viscosity and shear stress. Thermogravimetric analysis (thermal gravimetric analysis) determined PCL decomposition and the final HA content in the scaffold. Morphological properties, including porosity, pore size, and pore distribution, were evaluated using microcomputed tomography (micro-CT), while field-emission scanning electron microscopy imaged scaffold surface and cross-sectional morphology. Mechanical tests (compression and tension) assessed the scaffold strength. In vitro assays with MC3T3-E1 preosteoblast cells measured cell viability and alkaline phosphatase enzyme activity in uniform and FGSs with 10% and 20% HA content. Results confirmed that the achieved porosity levels provided sufficient strength and supported effective cell proliferation. Cell culture results demonstrated that uniform scaffolds with 10% HA promoted osteogenesis with slow cell proliferation, whereas FGSs with 20% HA promoted both proliferation and osteogenesis of preosteoblast cells. Overall, the structural, compositional, and biological characterization indicated that both uniform and FGSs provide suitable environments for bone tissue regeneration, with functionally graded scaffold morphology potentially offering a favorable environment for cell response.

## 1 Introduction

Bone tissue, distinguished by its mineralized composition, possesses a sophisticated hierarchical structure that undergoes daily remodeling.^1^ To successfully promote cell proliferation and osteogenic differentiation, an ideal scaffold for bone-tissue regeneration should possess optimal mechanical properties, including stiffness, pore diameters, surface topology, and load-bearing capacity.^2^ Additionally, the scaffold should be biocompatible. To imitate the mechanical characteristics of the host tissue for appropriate load transmission, the stiffness of the scaffold is among these sets to match the stiffness of human cancellous bone (elastic modulus between 0.1 and 2 GPa) or cortical bone (elastic modulus between 15 and 20 GPa). Additionally, by enabling cells to perceive the mechanical characteristics of the extracellular matrix, the scaffold’s stiffness can control how cells behave. Even though pore size and its impact on osteogenic cell differentiation have been well studied, there is still no consensus on the appropriate pore size.^2^ However, the following details are acknowledged: To form new bone, scaffolds with pore diameters of at least 100 μm are preferred, whereas 200–400 μm is the best range for the growth and regeneration of bone tissue. Pore sizes more than 500 μm in diameter have been demonstrated to cause a decrease in surface area, which is linked to a decrease in cell adhesion and a low degree of cell-to-cell contact. These factors have a negative impact on proliferation and the subsequent osteogenesis that leads to the production of bone.

Within the realm of scaffold fabrication, various methods have been proposed for both 2D (two-dimensional) and three-dimensional (3D) bone scaffolds.^3^ These methods, including solvent casting,^4^ particulate leaching,^4^ gas foaming,^5^ and freeze-drying,^6^ offer distinct advantages in crafting scaffolds to meet specific needs. However, additive manufacturing (AM) techniques^7^ relying on the use of 3D printing of resin, powder, filament, or ink materials^7,8^ such as stereolithography (SLA), selective laser sintering, fused deposition modeling (FDM), and electron beam melting can obtain not only customized external shape but also controllable porous internal structure for scaffolds, both of which are of great importance for repairing large segmental bone defects. More specifically, 3D printing is an AM technique that allows the fabrication of modular and patient-specific scaffolds with high structural complexity and design flexibility.^7^

Given the inherent structure of native bone, which includes a highly porous spongy core and a dense compact shell featuring multifunctional architecture with various pore sizes, the primary emphasis has been on achieving an optimal structure that mimics the multifunctionality found in bone structures.^9^ Recent advancements in bone tissue engineering have addressed the intricate structural and morphological changes observed in native bone, particularly the gradual alterations in pore distribution throughout its cross-section.^10^ A cutting-edge solution to this challenge involves the development of 3D-printed bone tissue scaffolds through the implementation of functionally graded scaffolds (FGSs) with tailored gradient properties such as material composition or pore distribution to functionalize biological^11^ or mechanical responses.^12,13^ More specifically, Nowicki et al. demonstrated that both mechanical properties and cell performance, such as human mesenchymal stem cell adhesion and proliferation, improved in the biphasic graded pore structure (200–300, 750, and 1450 μm spacing) of FDM-based 3D bioprinted and nanocrystalline hydroxyapatite scaffolds when compared to homogeneously distributed porous and nonporous structures.^11^ Most existing work, however, falls into the second category, where the role of various bioinspired gradient designs (using polyurethane acrylate ink to produce elastomer material gradients of a human intervertebral disc structure) is investigated on the local and global mechanical behavior of materials such as stiffness, strength, and strain at failure^12^ or on methods to control pore size to effectively generate porous scaffolds (triply periodic minimal surface (TPMS), Ti6Al4 V ELI powder grade 23) with tailored mechanical properties.^13^ In most existing studies, FGSs have been designed by taking inspiration from the inherent structure of the human bone, as emphasized in multiple research studies discussed in review papers.^10,14^ Current synthesizing techniques used to produce FGMs for load-bearing orthopedic applications and their mechanical, structural, and biological behavior have been presented to demonstrate the potential of FGMs.^10^ Another study presented one of the earliest attempts reporting the 3D printing of a porous TPMSs (TPMS)-based design, a special class of FGS, using ceramic materials, namely alumina.^14^ Their results showed reduced compressive modulus comparable with that of native bone and hence could potentially be adopted for bone implant design to mitigate the stress-shielding effect. Similarly, due to TPMS-based geometry’s ability to achieve gradient scaffolds in multiple patterns with continuous topology and interconnectivity, Liu et al. showed that cell-size gradients can adjust the surface area and pore size of Ti-6Al-4 V (ASTM grade 5 Titanium, alpha beta titanium alloy) scaffolds without relative density alteration, resulting in superior strength and comparable elastic modulus with cortical bone in their research by using different TPMS unit cell microstructures such as Gyroid (pore size approximately 3.5–5.25 mm), diamond (pore size approximately 3.5–4.25 mm), and gyroid-diamond (pore size approximately 3.75–4.0 mm) in their scaffold designs.^15^ It was demonstrated that the use of TPMS geometries effectively allowed gradients in multiple patterns that are comparable to natural tissue regarding both continuous topology and interconnectivity. At the same time, the porous surface area and pore size could be controlled via tuning the gradient in cell size without changing the relative density, hence stabilizing the effective modulus and strength, respectively. Experimental data showed that both gyroid and diamond structures possess a superior strength (152.6 and 145.7 MPa) and comparable elastic modulus (3.8 GPa) with natural cortical bone.

Dubey et al. provided a detailed review of functionally gradient materials (FGM) for bone regeneration, potential challenges, and characteristics.^10^ In addition to the pore size control, the use of inorganic FGM production, the utilization of functional grading either as bulk or coating, and the need for the development of a technique that allows the production of biodegradable metallic materials were stated.^10^ Zhang et al. used SLM to investigate the spatial variation in bone tissue scaffolds and its relationship to mechanical characteristics and permeability.^16^ As a result of their observations, they revealed that rather than gradient variation, the apparent porosity was the major determining factor of permeability.^16^ The permeability of gases and fluids in tissue scaffolds depends on the presence and characteristics of porosity (size, type, and distribution) since they create a pathway for those materials to be distributed within the scaffold. Proper distribution of such resources creates an environment for cells to proliferate, differentiate, and colonize, leading to tissue regeneration.^17^ Furthermore, a functional gradient can be used to modulate the structure’s permeability, which is a key factor in forecasting how cells will behave in scaffolds.^18^ Since the interior architecture of the pores directly affects the biological efficacy of lattice structures, it is feasible to anticipate cell activity during the lattice structure design phase by analyzing liquid permeability. Using a functional gradient to control the interior pore structure locally alters the scaffold’s permeability, which directly impacts the filtration rate and, consequently, the rate of chemical and biological processes. In another study, Zhang et al. showed that graded TPMS designs in the form of heterogeneous FGSs constructed by skeletal and sheet TPMSs they proposed showed superior mechanical stability, and energy absorption efficiency was enhanced by 3.0–79.0% and 2.6–16.8% compared to uniform skeletal and sheet TPMS, respectively.^19^

Upon revisiting the critical attribute of scaffolds, namely, porosity, several studies have been dedicated to exploring the impact of scaffold pore size and distribution on biological performance, in addition to their mechanical performance, with a particular focus on their role in bone regeneration. Findings from these studies indicate a correlation between the pore size and the scaffolds’ ability to support cell growth and proliferation.^20,21^ Consequently, a predominant theme in studying FGSs has been the investigation of pore gradients within their structures, revealing a notable enhancement in cell proliferation rates in pore functionally graded Ti6Al4 V scaffolds, which inherently are not biodegradable like typical scaffolds but biostable,^22^ as well as improved alkaline phosphatase (ALP) activity and the upregulation of osteocalcin and osteopontin in biodegradable materials such as poly(ε-caprolactone) PCL scaffolds.^23^ The latter study demonstrated that the heterogeneous pore sizes in gradient and fiber offset PCL scaffolds significantly improved the osteogenic potential of osteoblasts and hence may provide superior outcomes in bone regeneration applications. In the majority of existing work, pore gradients analyzed refer to macrosize pores between the struts^13−15,19,21,22^ with no dual porosity or the use of different techniques to obtain scaffolds with double porosity within uniform macropores.^24,25^ However, to the best of our knowledge, no study exists delving into or achieving an understanding of the significance of multiscale pores where simultaneously micro- and gradient macroporosity exists. The latter refers to pores between the extruded filaments through 3D printing, and the resulting scaffolds in this study employ design principles of FGSs. Inspired by this insight, our study centers on 3D printing of composite polycaprolactone (PCL) and hydroxyapatite (HA) FGSs and examination of their performance in comparison to uniform scaffolds, where the uniformity refers to the uniform size of the macropores between the struts in the scaffold/grid structure.

**Figure 1:**
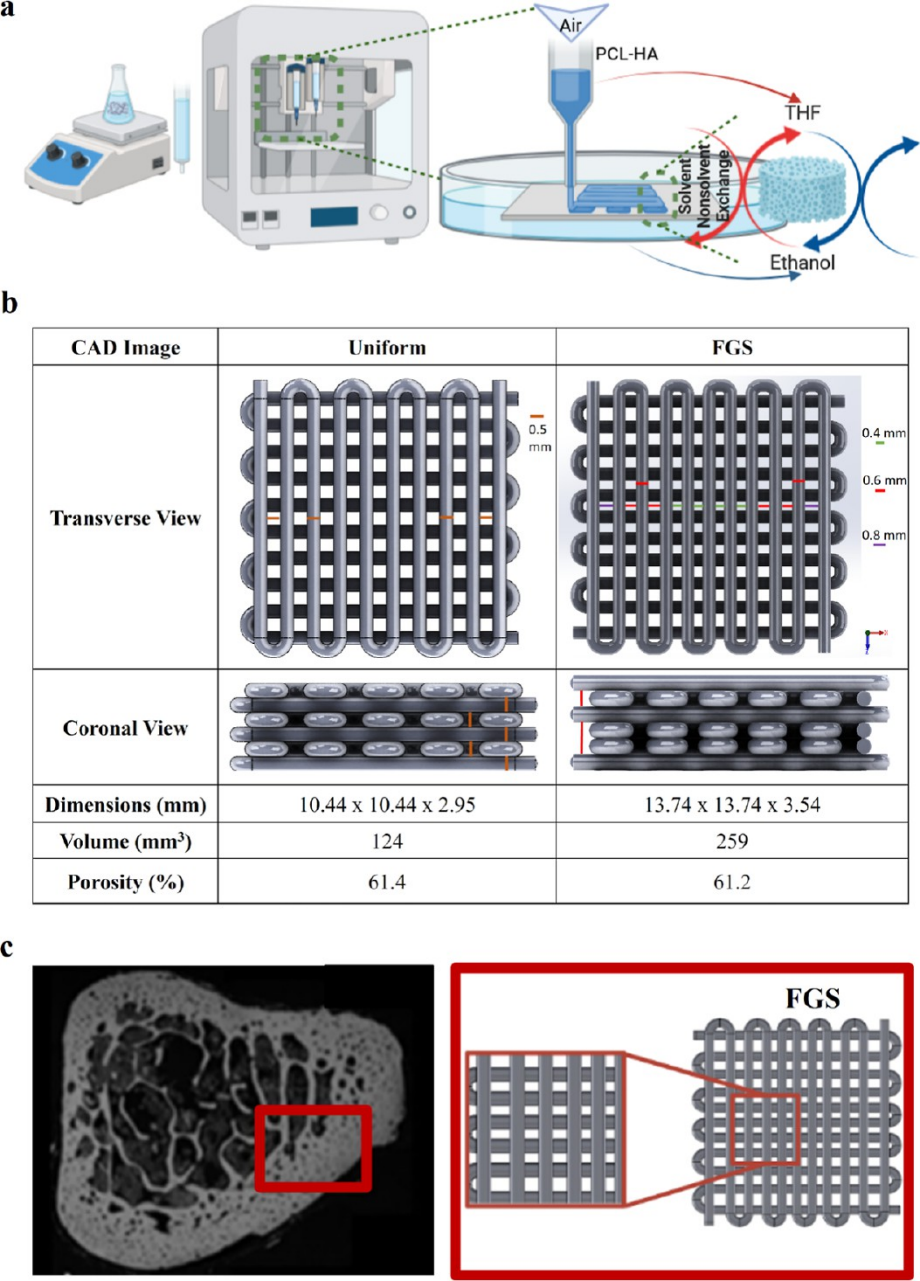
(a) Integration of the 3-D printing process with the NIPS method. (b) CAD design showing properties of uniform and FGSs. The uniform scaffold was designed to have a 0.5 mm distance between the struts everywhere, whereas FGS starts from 0.4 mm and gradually increases up to 0.8 mm from the center to edges on transverse view. The uniform scaffold has a uniform slicing height of 0.6 mm between the layers. FGS has a gradient distance between layers (in height/coronal view) of 0.6 mm and 1.2 mm as shown. (c) Bone tissue’s morphological characteristics through CT scans revealing its internal structure featuring varying levels of porosity^41^ and the FGS model to mimic natural bone tissue by incrementally increasing pore size.

Distinguishing itself from previous research,^11−15,22−25^ our exploration focuses on FGSs characterized by multiscale porosity, i.e., scaffolds with spatially varying macropores between struts and the simultaneous existence of micropores within struts. It is also noted that the grading of macropore size displays a variation in both radial and longitudinal dimensions. The main motivation in designing the FGS displaying a macroporosity gradient was to mimic the inherent spatial pore variation in a real bone (Figure 1C). The multiscale porosity is a feature achieved using nonsolvent-induced phase separation (NIPS), which is a widely employed technique in membrane fabrication involving the separation of a solid phase from a polymer solution.^26−28^ Notably, it maintains its advantage for thicker substrates (800–900 and 1600–1800 μm thickness), making it particularly valuable for applications in bone tissue engineering, as was reported earlier.^29,30^ While the combination of NIPS with 3D printing is a technique with substantial potential, literature reveals that very few studies have explored this synergy, and those that have focused exclusively on producing uniform scaffolds^31^ with limited ability to mimic the actual bone structure. In our current study, we utilized NIPS integrated into 3D printing to produce both uniform and FGSs. This approach operates on dual scales of porosity: microscale achieved via NIPS and macroscale using 3D printing in both uniform structures and FGSs with varying HA content.

The primary goal of this study was to examine the influence of both macropore gradient morphology and HA content within 3D bone tissue scaffolds, including micropores, on both mechanical and biological responses. Crafted using a solution-based 3D printer and incorporating 10% and 20% HA alongside PCL, these scaffolds featured uniform and macropore gradients in the radial and longitudinal directions.

The resulting PCL/HA FGSs showcased a unique multiscale porosity achieved through the NIPS technique, while the multidimensional grading was achieved through its integration to 3D printing. To assess mechanical properties, tension and compression tests were conducted because off-centric forces create a bending moment in the bone, leading to a distribution of tensile and compressive stresses; these are critical in imitating bone loading and hence were analyzed in evaluating the produced scaffolds. Structural analysis involved micro-computed tomography (Micro-CT) analysis, and scanning electron microscopy (SEM) imaging providing detailed insights into scaffold architecture. Additionally, for biological characterization, in vitro experiments explored ALP activity (ALP) and immunocytochemistry employing the MC3T3 cell line and confocal imaging elucidating the biological responses of the scaffolds.

## 2 Materials and Method

### 2.1 Preparation of PCL/HA Suspension

PCL pellets (*M*_n_: 80,000 Da; Sigma-Aldrich, St. Louis, MO, USA) were dissolved in tetrahydrofuran (THF) at 40 °C through a magnetic stirrer and progressively added to prepare 15% w/v PCL suspensions. In the literature,^25,32,33^ PCL concentration used for the fabrication of scaffolds using the NIPS method ranged from about 10%–22% for producing scaffolds. After the PCL pellets were completely dissolved, various HA (<200 nm; Sigma-Aldrich, St. Louis, MO, USA) (see Supporting Information Figures S1–S3) contents (10% and 20%) were gradually added to the PCL suspension until a homogenized HA dispersion was obtained. Prepared suspensions were transferred to cartridges of the 3D printer (Figure 1A). Despite formulating three polymer suspensions in this study, 20% PCL with 10% HA; 15% PCL with 10% HA; and 15% PCL with 20% HA, only the latter two suspensions were presented. This choice was justified and elaborated upon in the Results and Discussion sections. For ease of reference, these groups will be denoted as PCL20HA10, PCL15HA10, and PCL15HA20, respectively. All percentages are based on weight to weight (% wt/wt).

### 2.2 Geometry of Scaffolds

The scaffolds were designed by SolidWorks 2018. The dimensions for uniform and FGSs are 10.44 mm × 10.44 mm × 2.95 and 13.74 mm × 13.74 mm × 3.54 mm, respectively (Figure 1B). The theoretical overall porosities for uniform and FGSs are around 61% (Figure 1B). The spacing between the struts in the FGS is inherently nonuniform and therefore differs from the dimension of the uniform design to maintain similar pore size and strut sizes. This variation results in distinct overall dimensions in the *XY* plane for the two designs. However, for both designs, a comparable porosity value of 61% based on their morphological design parameters was maintained by tuning the gradient in the layer height of the scaffold structure in the *Z* direction. SolidWorks was used to calculate the overall design porosity of uniform and FGSs. Both porosity percentages for each design are provided in Figure 1B. After forming the CAD design, the geometries are saved in standard template library format. Then, the gcode was formed after slicing the stl by using HeartWare software.

### 2.3 Integration of 3D Printing and the NIPS Method

3D uniform and FGS bone scaffolds were printed via an incredible solution-based 3D printer (CELLINK AB, Gothenburg, Sweden). The prepared composite suspension was placed into the cartridge that is durable with our suspension, and the printing of the suspension was performed via a pneumatic system. The standard blunt metal needle nozzle was used as the suspension, which contained tetrahydrofuran (THF) as the solvent. A 25G nozzle (0.4 mm diameter) was selected as the optimal size for printing scaffold struts with the intended thickness while remaining within the pneumatic pressure limits of the printer. The suspension was at room temperature after preparation and during printing, which was carried out under the hood. Right before printing, a well-stirred suspension was loaded into the cartridge, and printing was carried out continuously under optimized printing conditions to prevent any clogging in the nozzle. When clogging occurred, we changed the nozzle to a new one. The nozzle was submerged in an ethanol bath during the printing (Figure 1A) process to eliminate the interaction of the suspension-air during the extrusion. To integrate 3D printing and the NIPS method, the key factors, such as pneumatic printing pressure, printing speed, and slicing thickness, were fine-tuned and optimized to ranges of 150–200 kPa, 10–15 mm/s, and 0.1–0.2 mm layer height, respectively. According to the viscosity of the suspension, these settings were tuned before printing.

### 2.4 Rheological Characterization for Printability

The viscosity of the printing suspension is the first factor to consider in the successful printability. The viscosity of the suspensions of PCL15HA10, PCL15HA20, and PCL20HA10 3D tissue scaffolds was determined using a rheology test. At room temperature, amplitude scanning and flow tests were performed using an Anton Paar MCR302 Rheometer (Anton Paar, Austria) and parallel plates of 25 mm (PP25) with a gap size of 0.5 mm. The amount of suspension used was around 0.25 mL, which was enough to cover the volume under the cone plate, and the excess material was cleared from the vicinity of the cone-plate. After each sample was loaded, a thin layer of low-viscosity paraffin oil was applied to shield the samples from moisture adsorption along the outer edges of the plates. Due to the high volatility of THF in suspensions, covering equipment was employed during the test to prevent evaporation. In the amplitude test, the frequency was set to 1 Hz, and the strain was changed from 0.01 to 300%.^32^ For the flow test, the viscosity of the suspensions was calculated by finding the linear region from the amplitude sweep test, choosing the value that corresponded to the linear area and then using that value (1%). Three replicates for each suspension were measured. The graphs and data of viscosity and shear rate versus shear stress were collected.

### 2.5 Morphological Analysis with Micro CT and SEM

#### 2.5.1 Micro-CT

Micro-CT (SkyScan 1172; Bruker, Belgium) was used to assess the morphology of the scaffolds in terms of the total porosity and pore size distributions. After the scanning is complete, the object’s 3D structure was rebuilt from 2D slices using the NRecon program. The DataViewer software was used to generate morphological images of specimens for a variety of cross-section slices. The CTAn software (SkyScan) was used for 3D morphological analysis. A round-shaped region of interest was applied to these 101 chosen slices to provide a similar volume of interest. It is noted that the calculations were repeated with different numbers of slices, but the results did not produce significant changes due to the uniform distribution of pores. Hence, the process was continued with a chosen set of 101 slices to optimize the computation time, as increasing the number of slices significantly extends the 3D analysis duration without yielding substantial improvements in accuracy. Since the porosity induced by phase separation in the specimens is uniformly present and well-distributed, only a single sample was scanned for analysis. However, calculations were based on at least 3 sets of multiple sections and data sets from that sample to ensure comprehensive and reliable results. To match the original CT image to a binary image, the lower and higher grayscale threshold values were set to auto and 255, respectively. Optimized scanning parameters for microporosity detection in scaffold struts included a camera pixel size of 8.75 μm, a resolution of 2672 × 4000 pixels, 0.97 μm magnification, 40 kilovolt (kV) source voltage, 1490 ms exposure time, and a 0.25° rotation step. For scaffold visualization, parameters were set as follows: 8.75 μm camera pixel size, 1336 × 2000 pixels resolution, 6 μm magnification, 60 kV source voltage, 145 ms exposure time, and a 0.4° rotation step during scanning.

#### 2.5.2 SEM

Field emission scanning electron microscopy (FE-SEM) (Zeiss Leo Supra VP 35 field emission) was used to examine the top surface and cross-sectional porosity of 3D scaffolds as well as the cell morphology on the scaffold surfaces. For each type of geometry, one scaffold was used, and the cross sectional and surface images were taken from the same scaffold. Multiple areas on each scaffold were examined, both from the surface and cross-sectional views, and a representative image that characteristically presented a common observation among all was chosen. It is also noted that the same region was imaged using different magnification ratios to analyze the effect of that particular location, with respect to the scaffold morphology.

For the FE-SEM preparation of the cell-cultured scaffolds, in a 0.1 M potassium phosphate buffer, MC3T3-E1 cells are fixed to the surface for 2.5 h using a prepared solution of 2.5% glutaraldehyde. Following that, cells were dehydrated with a graded ethanol series (10 min of 35%, 50%, 70%, 80%, 90%. and 95% v/v and 15 min of 100% v/v) and then in a graded ethanol/1,1,1,3,3,3-hexamethyldisilazane (HMDS) (Acros Organics) solution series (10 min of 50%, 60%, 70%, 80%, and 90% v/v) and after 100% HDMS air-dried for HMDS removal.

To prepare for cross sectional image analysis, specimens were exposed to liquid nitrogen before slicing samples with pliers. Then, these samples were gold–palladium sputtered for 135 s using a Denton vacuum sputtering apparatus. The accelerating voltage of 3.0 kV was used for scanning the scaffold surfaces.

### 2.6 Thermogravimetric Analysis of Scaffold Composition

Thermogravimetric (TGA) analysis was used to estimate the compositional ratios of the polymer and bioceramics. Scaffolds were heated using nitrogen gas at a rate of 10 °C per minute from 30 to 500 °C. 2.473 g of the sample was used for the measurement. Finally, residual HA was calculated by tracking the mass loss of the sample.

### 2.7 Mechanical Characterization

The structural stability of both 3D-printed uniform and FGSs was examined using a tensile and compression test by using a MARK-10 (Mark-10 Corporation, Copiague, New York, USA) equipment with a 1 kilo Newton load cell, and 3 samples were tested for each mechanical test at room temperature. Tensile tests were carried out at 1 mm/min, and compression tests were carried out at 2 mm/min. Pneumatic grips were used during the test. The dimensions and shape of specimens were based on 3D-printed uniform and FGS geometry and size, as shown in Figure 1B. For the compression tests, scaffolds with 8.6 mm × 9.3 mm (*XY*) cross sectional area (average 80 mm^2^) and for the tension tests, scaffolds with 2.6 mm × 8.6 mm (*ZX*) cross sectional area (average 22 mm^2^) samples were used, respectively. These dimensions were also used for performing stress calculations. The number of samples used for each test per scaffold type was 3. Tests were carried out at room temperature. After the stress–strain measurement plots were obtained, the compressive and tensile moduli were calculated based on the linear elastic region, with data chosen corresponding to strain values up until the 0.2 strain limit. The slope of the linearized stress strain data was used in the final moduli estimations. Also, the absorbed energy values were calculated based on the area under the stress–strain measurement curves.

### 2.8 Biological Experiments and Characterizations

#### 2.8.1 Assessment of Cell Culture Conditions

Cell culture studies were carried out with the MC3T3-E1 Subclone 4 cell line (ATCC CRL-2593) by using minimum essential medium, Alpha Modification (Alpha-MEM) (Gibco and Florabio) supplemented with 10% fetal bovine serum (Pan Biotech) and 1% penicillin–streptomycin solution (Pan Biotech) as a growth medium. Cells seeded on either tissue culture plates or tissue scaffolds were incubated at 37 °C, 5% CO_2_ humidified chamber until 70% confluency was achieved or appropriate time intervals.

Following the optimization studies with (3-(4,5-dimethylthiazol-2-yl)-5-(3-carboxymethoxyphenyl)-2-(4-sulfophenyl)-2*H*-tetrazolium) (MTS), ALP assays, and Alizarin Red staining, Mineralization medium and cell seeding numbers were chosen (see Supporting Information Figures S4–S6, respectively). More specifically, as was demonstrated in earlier work,^29^ for thin-film scaffolds (1 or 2 mL thickness) fabricated using NIPS, with 10% PCL and changing percentages of HA content (0%, 10%, 15%, and 20%), were seeded with 3 different cell concentrations of 20.000, 10.000, and 50.000 MC3T3-E1 cells to select the most suitable cell seeding number. For completeness of the presented work demonstrated for 3D functionally graded FGS and uniform scaffolds in this paper, information for optimization studies is added into the Supporting Information in Figures S4–S6. As a result of this optimization, 20,000 cells were used throughout the cell culture experiments. The optimization studies of MTT and Alizarin Red S assay were conducted in 96-well plates (127.71 mm × 85.43 mm × 14.70 mm), while ALP was done in 12-well plates, only with cells seeded on the previously mentioned well plates. These experiments were conducted to decide on the optimized cell number and Mineralization Medium composition for in vitro experiments. Thus, the cells were not differentiated in osteoblasts, but their differentiation behavior was further investigated both in optimization studies and the following in vitro experiments, as provided in Section 2.8.5.

It is also noted that since the optimization experiments regarding mineralization medium and ALP and Alizarin Red assay were conducted only using seeded cells and not scaffolds, this part of the optimization study was independent from HA presence or percentages. The information for optimization studies are added to the Supporting Information in Figures S4–S6. All the assays had 3 replicates of each condition (for days, HA percentages, and medium type).

#### 2.8.2 Cell Seeding and Culturing on 3D PCL/HA Scaffolds

Scaffolds were washed with sterile 1× Dulbecco’s phosphate-buffered saline (DPBS) and then placed in 70% ethanol for 1 h. Ethanol was removed, and scaffolds were transferred to a new sterile dish. After scaffolds were rinsed with sterile 1× DPBS two times for 30 min, they were sterilized under ultraviolet (UV) light for 1 h on each surface. Sterile scaffolds (PCL15HA10 and PCL15HA20 uniform scaffold dimensions are 10.44 mm × 10.44 mm × 2.95 mm, while PCL15HA10 and PCL15HA20 FGS scaffold dimensions are 13.74 mm × 13.74 mm × 3.54 mm, respectively), were seeded with MC3T3-E1 cells at a concentration of 5200 cells/cm^2^ in 100 μL growth medium. For each scaffold type and time point (days), 3 replicates were used. Cell seeding was performed while keeping the scaffold in a hydrophobic culture dish (35 mm confocal dish) to ensure that the cells adhered to the surface of the scaffold but not to the culture dish. Scaffolds with seeded cells were cultured for 2 h at 37 °C within an incubator. This ensured that the cells adhered to the surface of the tissue scaffold, but not to the culture dish. The number of adhered cells was not checked after the incubation; however, an MTS assay was carried out on a weekly basis to observe the increase in the relative cell number. The scaffold samples were then moved from the culture dish to the 12-well cell culture plate and immersed in growth media. After 3 days, the growth medium was replaced with a mineralization medium to initiate mineralization.

#### 2.8.3 Assessment of Cell Metabolic Activity

The cell viability on 3D scaffolds was assessed at, 14, 21, and 28 of culture by using the MTS (Abcam) kit. Cell seeding was carried out with growth medium. After 3 days, the culture media was changed with the mineralization medium and renewed every 2–3 days. Briefly, scaffolds were removed from the media and placed onto a clean 24-well plate. 500 μL of a 10% MTS-containing solution was added to each well and incubated for 4 h at 37 °C and 5% CO_2_. 200 μL of reaction solution of each sample were transferred to a 96-well plate as triplicates, and absorbance was measured using a plate reader (BioRad) at 490 nm.

#### 2.8.4 Assessment of ALP Activity

Cells were cultured for 28 days on uniform and FGSs containing 10% and 20% HA and 15% PCL. Cell seeding on scaffolds were carried out by growth medium, and it was changed with mineralization medium after 3 days culture. ALP activity of the cells was measured at culture days 7, 14, 21, and 28. Cellular ALP activity was measured from the cell lysate. Briefly, cells were collected from scaffolds by incubating with 0.25% trypsin (ethlyenediaminetetraacetic acid) (Trypsin–EDTA) solution (Pan Biotech) for 20 min. Cell pellets were suspended in 300 μL of radioimmunoprecipitation assay buffer (RIPA) containing protease inhibitors. All samples were vortexed five times, each for approximately 10 s. After being centrifuged at 10,000*g* (10 000 times gravity force) for 10 min at 4 °C, the supernatants were transferred to new tubes. 2 μL of each sample was used to reduce 2 μL of *p*-nitrophenyl phosphate (pNPP) in 100 μL total reaction volume in 45 min. Reduced pNPP was followed by measuring the absorbance at 405 nm. Total protein concentration was measured for each sample by using a Bradford assay and using results to normalize the ALP activity. This allowed the prevention from the potential influence of the HA content in ALP assay measurements.

#### 2.8.5 Phalloidin Staining of 3D PCL/HA Scaffolds

Using 4′,6-diamidino-2-phenylindole (DAPI) and phenylindole staining, immunocytochemistry was executed to evaluate the morphology and proliferation of the cells. Phalloidin Alexa Fluor 546 (Invitrogen) and Fluoroshield with DAPI (Sigma) were used in staining investigations to look at cell distribution and morphology, as described in the following paragraph.

For staining, culture media was collected, and samples were rinsed three times in sterile 1× DPBS and fixed with 4% paraformaldehyde (PFA) solution for 30 min at room temperature. Fixation solution was removed and rinsed with sterile 1X DPBS before adding 0.1% Triton X-100 containing phosphate buffered saline (PBS) to achieve cell permeability and incubated for 15 min at room temperature. Finally, samples were blocked with a 45 min incubation in 1% bovine serum albumin-containing PBS at room temperature. The samples were then incubated in the dark for 1 h at room temperature, according to the manufacturer’s instructions, with phalloidin Alexa Fluor 546 (Invitrogen) diluted to 1:400 in PBS. At the end of the incubation, the antibody solution was collected, and the sample was washed in sterile 1× DPBS. Samples were placed on a confocal dish, and a drop of Fluoroshield with DAPI (Sigma) was used on them. After 5 min of incubation in the dark, observation was made under a confocal microscope (Zeiss, Axio Observer).

### 2.9 Statistical Analysis

Statistical analysis was conducted by using one-way analysis of variance (one-way ANOVA) with Tukey’s multiple comparison test in GraphPad (version 5, California, USA). Data are expressed as the mean ± the standard deviation (SD). Differences were considered statistically significant at *p* < 0.05.

## 3 Results

### 3.1 Rheological Characterization for Printability

**Figure 2:**
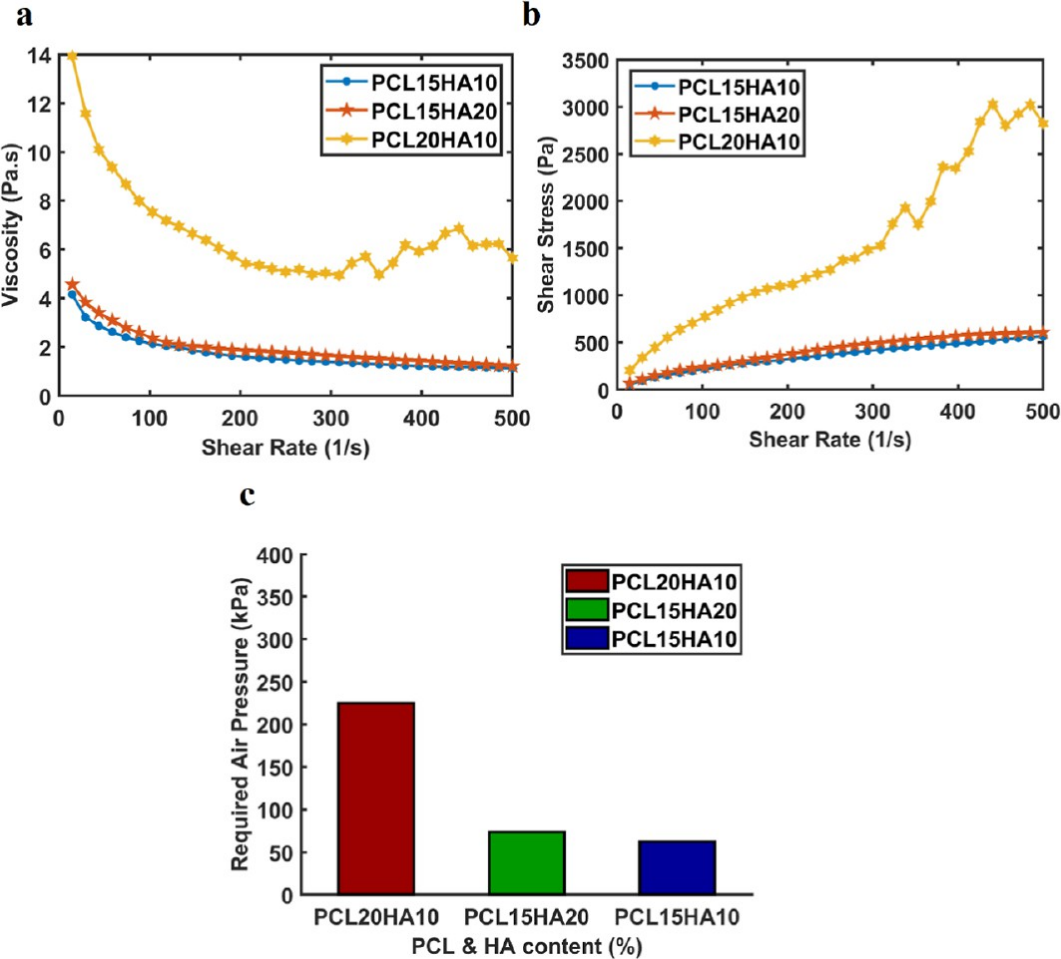
(a) Viscosity vs shear rate plot, (b) shear stress vs shear rate plot, and (c) required quantities of air pressure in a 3D printer obtained using shear stress–strain–viscosity relation.

Viscosity-shear rate and shear stress-shear rate results for various PCL/HA suspensions were measured and are displayed in Figure 2A,B, respectively. Viscosity decreases with increasing shear rate/stress; thus, those suspensions show shear thinning behavior, as depicted in Figure 2A. We investigated the effect of hydroxyapatite (HA) concentration on viscosity by comparing PCL15HA10 with PCL15HA20 and similarly assessed the influence of polymer concentration by examining PCL15HA10 against PCL20HA10. Both HA and polymer content resulted in increased viscosity, making the ink more resistant to flow and necessitating higher air pressure to overcome this viscosity for printing. Additionally, it was expected that the polymer content in the ink would have a more significant and dominant influence on viscosity. As anticipated, the suspension containing 20% PCL with 10% HA (w/w) (PCL20HA10) demonstrated the highest viscosity, aligning with expectations due to its higher polymer concentration. Comparing the PCL15HA20 and PCL20HA10 suspensions, the results showed a substantial increase in viscosity with higher PCL concentration, resulting in an elevated resistance to suspension flow. Consequently, suspensions with higher PCL concentration required greater applied pressure. Furthermore, it was observed that the viscosity of the PCL15HA20 was slightly higher than that of the suspension with 10% HA. However, the effect of increasing HA concentration was not as pronounced as that of polymer concentration. This rheological analysis confirmed that the addition of PCL and/or HA leads to an increase in viscosity, consequently necessitating higher levels of air pressure for the printing process. In the context of 3D printing using a solution-based printer, PCL20HA10 required the highest air pressure, followed by PCL15HA20, and finally PCL15HA10 (as demonstrated in Figure 2C). From the shear stress and shear rate graphs in Figure 2B, the shear stresses corresponding to the same shear rate in the linear and stable regions were identified for all three suspensions. The associated air pressure required to print these suspensions using a blunt needle nozzle was also determined. Using this data, the pressure range was computed using eq 1(34) based on Newtonian laminar flow and is presented as a bar graph in Figure 2C. The shear stress value was extracted at a shear rate of 294 (1/*s*) for all three suspensions.
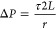
1

In eq 1, τ represents shear stress, *L* denotes the nozzle length (17 mm), and r stands for the nozzle radius (*d* = 0.45 mm). As demonstrated in Figure 2C, for the same sliding speed, the suspension containing 20% PCL and 10% HA, due to its high viscosity, necessitated the highest air pressure, which happened to be the maximum pressure limit of the used printer. To safeguard the printer, the applied air pressure should not exceed 350–400 kPa. This established a constraint on the compositional weight content ratio of PCL and HA for fabricated scaffolds. Consequently, after this assessment, the scaffold types for further analysis were designated as PCL15HA10 and PCL15HA20. PLC20HA10 was excluded from the study to work well below the maximum air pressure of the printer.

### 3.2 Morphological Analysis with Micro-CT and SEM

#### 3.2.1 Micro-CT

**Figure 3:**
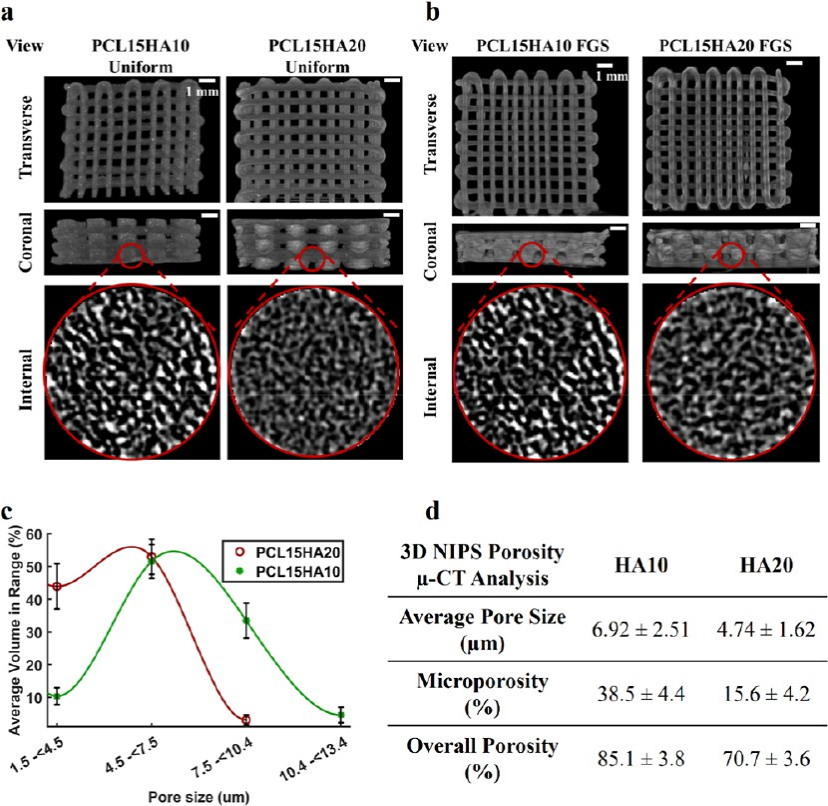
(a, b) Microcomputed tomography images of uniform and FGSs from coronal, transverse, and sagittal planes, respectively. Scale bars show 1 mm. (c) Micro-CT 3D microporosity analysis of printed scaffolds with different HA content (10% and 20% w/w). The *X*-axis refers to the pore size range in 3D, and the *Y*-axis refers to the percentage volumetric finding in the respective pore size range. Since the microstructures of the uniform and FGSs are the same due to the same fabrication method, the analyses were applied only to the struts of uniform 3D structures. (d) 3D Microporosity analysis listing average pore size, average microporosity due to NIPS in the printed filaments/struts regardless of printing design (uniform and FGSs), and overall porosity (design porosity + microporosity) of 3D scaffolds.

Microporosity images of struts are given in Figure 3A,B for uniform and FGSs, respectively. A selected section view of the relevant region used for the microporosity analysis of 3D samples is given in the bottom row of Figure 3A. The average pore size and overall porosity calculation in the relevant region are tabulated in Figure 3D. As shown in Figure 3A,B based on the internal views of uniform and FGSs, it is observed that uniformly dispersed microscale pores were formed at various HA concentrations. These micropores within 3D-printed scaffolds are expected to improve tissue regeneration by increasing the surface area after implantation at the fracture site. To comprehend and elucidate the morphological properties of printed scaffolds, it was imperative to grasp the impact of suspension viscosity on pore formation and porosity. Although there is no conclusive evidence suggesting an inverse relationship between viscosity and pore size based on rheology tests in Figure 2A, which indicate that PCL15H10 and PCL15HA20 exhibit similar viscosity values, micro-CT 3D analysis results revealed that PCL15HA20 produced smaller micropore sizes compared to HA10, likely due to the higher concentration of HA, which may have caused particle clumping and potentially resulted in smaller pore size and increased internal porosity. The microporosity value for PCL15HA10 was determined to be around 38.5 ± 4.4%, while that for PCL15HA20 was measured as 15.6 ± 4.2%, respectively. The overall scaffold porosity, encompassing both design porosity and internal microporosity, was calculated, taking microporosity values into account, for all struts of PCL15HA10 and PCL15HA20, regardless of scaffold morphology design (i.e., uniform vs FGS). This calculation revealed overall porosities of 85.1 ± 2.7 and 70.8 ± 2.6, respectively. Figure 3C shows that at least half of the pores in both PCL15HA10 and PCL15HA20 samples fall within the 4.5–7.5 μm range. In the smaller pore size range of 1.5–4.5 μm, PCL15HA20 exhibited a higher pore volume percentage of approximately 45%. Conversely, in the larger pore size range of 7.5–10.4 μm, PCL15HA10 showed a higher pore volume percentage, at around 35%, marking a notable difference from PCL15HA20. Additionally, while no pores were detected for PCL15HA20 in the 10.4 to 13.4 μm range, PCL15HA10 demonstrated some presence of larger pores, indicating that PCL15HA10 tends to form slightly larger pores compared to PCL15HA20. The microporosity of struts and overall porosity of uniform and functionally graded 3D-printed scaffolds for two HA concentrations were given in the Table in Figure 3D. Macroporosity (the distance between the struts of the scaffolds) of the functionally graded structure was also measured through micro-CT images. The majority of the pore size diameters in the *x*–*y* direction (transverse view), which is the plane displaying radial porosity, can be separated into mainly three groups: 450–500, 620–670, and 750–800 μm for 10% HA; 400–450, 600–620, and 650–750 μm for 20% HA, whereas the pore size in the *z* direction gradient (coronal view) can be grouped into pores with 450–550 μm (doubled pore size in design) and 250–350 (single pore size in design) μm, respectively.

#### 3.2.2 SEM

**Figure 4:**
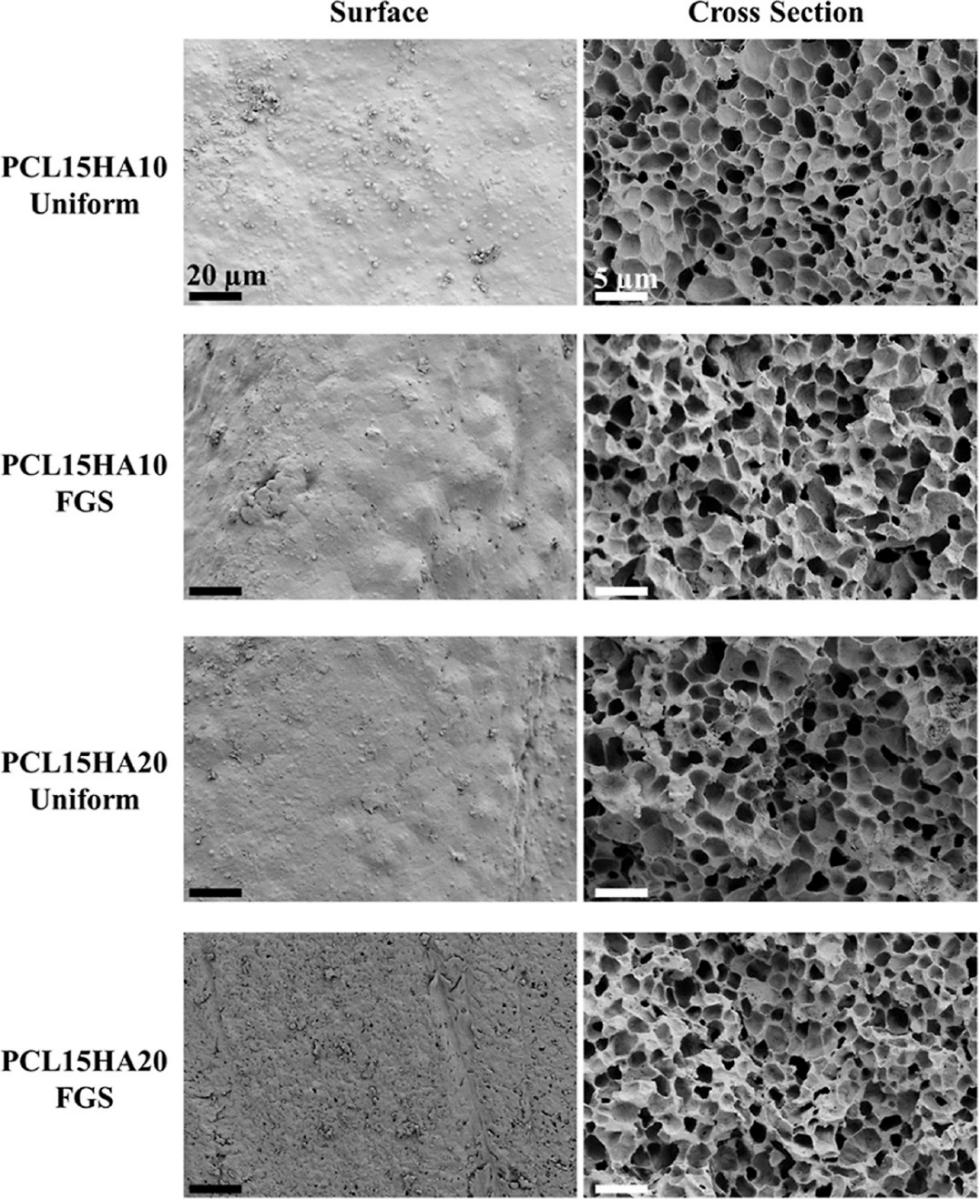
FE-SEM images showing surface and cross-sectional morphology (microporosity) of uniform and FGSs. All scaffold groups exhibited similar microarchitecture due to NIPS micropore formation mechanism. The gradient effect was not observed since it has a macroscale design (CAD) property. Scale bars show 20 and 5 μm corresponding to 2000× and 5000× magnifications with working distances of 9.7 and 10.4 mm for surface and cross-section views, respectively. A secondary electron detector was used with 3 kV of EHT.

Field emission scanning electron microscopy (FE-SEM) imaging was conducted to reveal surface porosity information for primarily a qualitative assessment of morphological surface features on both cross sections and the outer surface of 3D-printed uniforms and FGSs. Microporosity with honeycomb-shaped pores appears to be homogeneously distributed based on qualitative inspection of SEM images of the cross section for various HA contents (10%, 20%) (w/w) and geometries (uniform, FGS), as shown in Figure 4. The formation of “honeycomb-shaped” pores can be explained through thermodynamic principles involving energy minimization, phase separation, and material self-organization. In polymer systems, the formation of a honeycomb structure can also arise from phase separation due to thermodynamic incompatibilities between different polymer components or between the polymer and additives, such as hydroxyapatite (HA) in our case. During phase separation, two immiscible phases will segregate to reduce their interfacial energy. The more rigid or solid phase (e.g., particles of HA) will act as a framework, while the other phase (e.g., the polymer matrix) evaporates or solidifies. This leaves behind a regular structured network of pores in the form of a honeycomb. High-viscosity materials can inhibit the mobility of particles and solvents, promoting the formation of ordered structures such as honeycomb pores. Low-viscosity materials may form more random or irregular pores, while higher viscosity ensures more consistent, ordered phase separation and pore formation. Detailed surface and cross-sectional morphology of NIPS-based PCL scaffolds in the form of uniform film substrates were documented in our previous study.^29^ Notably, the surfaces of the printed scaffolds exhibited minimal to no porosity across all groups. The absence of surface porosity is likely due to the high shear exerted by the nozzle during the 3D printing process. In addition, there is no significant difference in surface porosity between the groups based on SEM image analysis. In an earlier study, it was demonstrated that surface porosity is present, particularly on the bottom surface of the film scaffolds.^29^ Despite the lack of surface porosity, it could be argued that internal porosity, with its ability to change the surface topography and roughness, could potentially lead to enhanced cell adhesion. A more detailed follow-up study would be needed to investigate the precise interrelationship between internal porosity and surface topography and its effect on cell adhesion. Nevertheless, the PCL15HA20 groups demonstrated slightly higher surface porosity compared to that of PCL15HA10. This difference could potentially be ascribed to the elevated concentration of HA particles near the surface.

### 3.3 Thermogravimetric Analysis of Scaffold Composition

**Figure 5:**
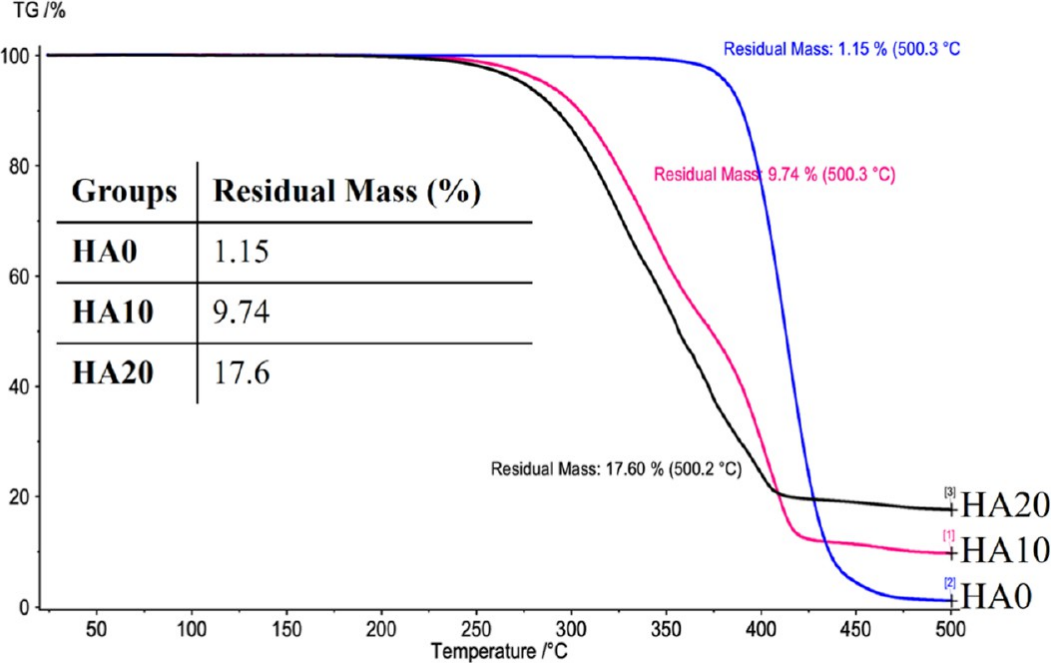
TGA of printed scaffolds with various HA contents (0, 10, and 20 wt %). Temperature-induced mass changes in PCL/HA scaffolds.

To assess the material composition, specifically the organic (HA)—inorganic (PCL) phase, thermogravimetric analysis (TGA) was performed, as shown in Figure 5. Thermal degradation (% change in mass) was observed, ranging from 250 to 500 °C. The initial decomposition temperature gradually increased with decreasing HA content. It was observed that PCL-HA scaffolds started to decompose earlier (at lower temperature) than pure PCL. However, pure PCL decomposed faster (with a steeper slope) after decomposition kicked off. The resulting residual content of scaffolds containing 10 and 20 wt % HA was determined to be 9.74% and 17.6% by weight, respectively. Therefore, the theoretical content of HA differed from the experimental results as follows: for 10 wt % HA suspensions, a 0.974% difference was observed, and for 20 wt % HA suspensions, a 2.92% difference.

### 3.4 Mechanical Characterization

**Figure 6:**
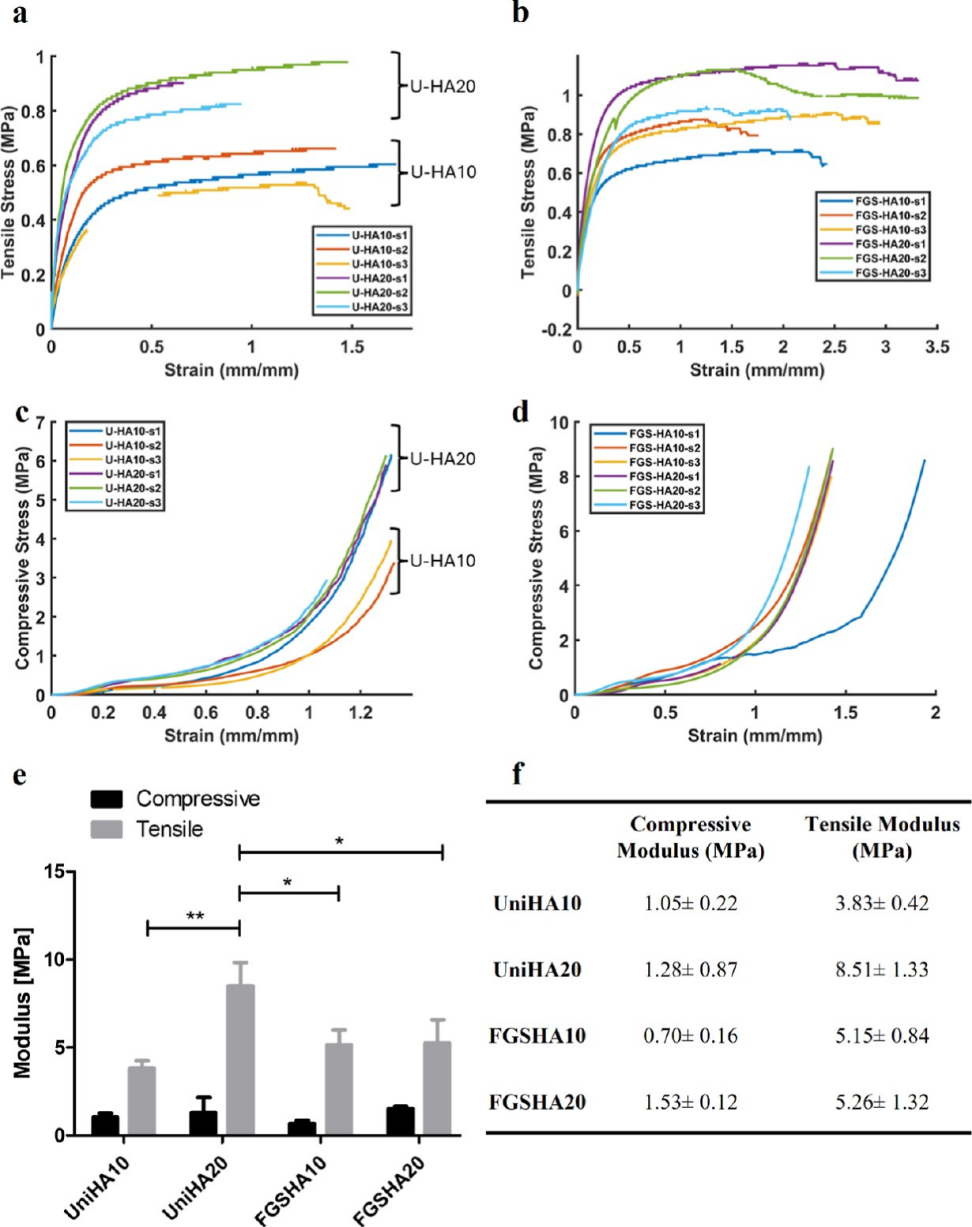
Mechanical testing results of HA10 and HA20 uniform and FGSs: Tensile stress vs strain curve for (a) uniform and (b) FGS scaffolds; compressive stress vs strain curve for (c) uniform and (d) FGS scaffolds; (e) bar plot and (f) table showing tensile and compressive moduli of uniform and FGSs with composition of HA10 and HA20.

To evaluate the mechanical properties of printed scaffolds, mechanical tensile and compression tests were conducted. Typical stress–strain curves and moduli were obtained when scaffolds were under tensile and compressive force and are shown in Figure 6. The moduli values are in the same range of orders of magnitude. An increase in the HA content led to a rise in the tensile modulus of uniform scaffolds. Among them, the uniform HA20 scaffolds exhibited the highest tensile modulus, surpassing that of their FGS counterparts (*p* < 0.05). Although not statistically significant, FGSs generally showed a slightly higher tensile modulus compared to uniform scaffolds. Due to the design, geometry of the samples, and the ductile nature of polymeric scaffolds, the compression test was less effective and accurate than the tensile test. As expected, the compressive modulus was lower than the tensile modulus, and no significant differences were observed.

Ultimate/maximum tensile strength from the stress–strain curve was found to be 0.6 ± 0.05, 0.9 ± 0.06, 0.83 ± 0.08, and 1.08 ± 0.1 MPa for the HA10Uniform, HA20Uniform, HA10FGS, and HA20FGS groups, respectively. Similarly, maximum compressive strength from the stress–strain curve was found to be 4.5 ± 1.19, 5 ± 1.45, 8.47 ± 0.34, and 8.67 ± 0.28 MPa for the HA10Uniform, HA20Uniform, HA10FGS, and HA20FGS groups, respectively. Maximum tensile strain was found to be 1.54 ± 0.12, 1.03 ± 0.34, 2.38 ± 0.48, and 2.34 ± 0.72 mm/mm for the HA10Uniform, HA20Uniform, HA10FGS, and HA20FGS groups, respectively. Similarly, maximum compressive strain was found to be 1.32 ± 0.05, 1.22 ± 0.1, 1.6 ± 0.25, and 1.35 ± 0.06 mm/mm for the HA10Uniform, HA20Uniform, HA10FGS, and HA20FGS groups, respectively. To find the absorbed energy up until the fracture point, the toughness values (the area under the curve of the entire tensile test stress–strain graph) were calculated as 1.77, 2.85, 0.87, and 0.83 MPa for the FGS PCL15HA10, FGS PCL15HA20, Uniform PCL15HA10, and Uniform PCL15HA20 samples, respectively. These results indicate that FGSs display a toughness value higher than that of uniform scaffolds in contrast to their tensile modulus values. Therefore, it can be argued that FGSs tend to absorb more energy than uniform scaffolds.

### 3.5 Biological Experiments and Characterizations

#### 3.5.1 Assessment of Cell Metabolic Activity

**Figure 7:**
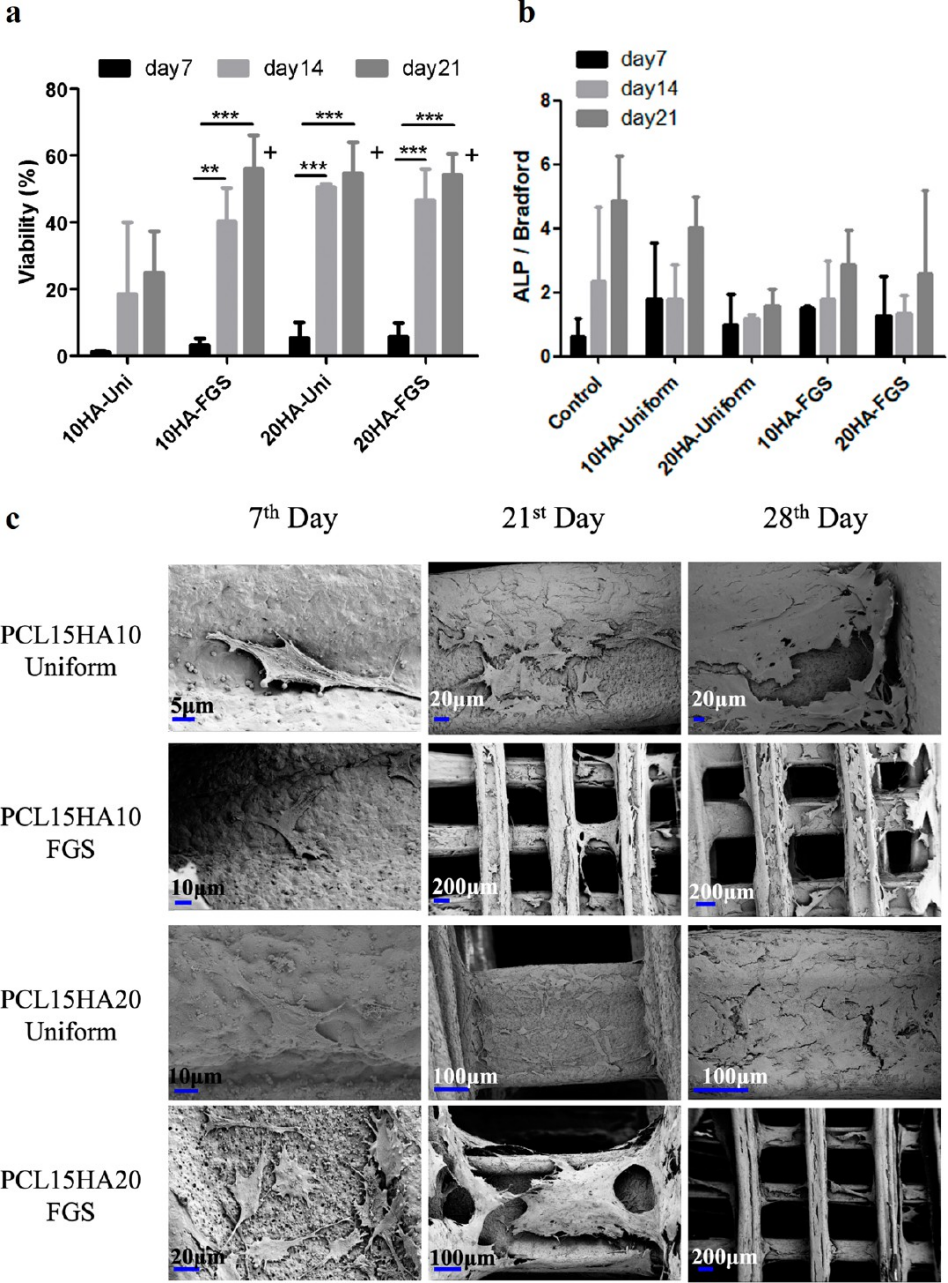
(a) Result of cell viability for 10% HA and 20% HA in architectures of uniform and FGSs cultured with cells on mineralization medium (MM). (b) Result of ALP activity for 10% HA and 20% HA in architectures of uniform and FGSs cultured with cells on MM. (c) FE-SEM images showing cell morphologies on uniform and FGSs with different HA contents at different time points. One-way Anova was used in statistical analysis. (SD ± St dev, *n* = 2). Plus-sign (+) shows a statistically significant difference between a group and the control group (PCL15HA10-uniform scaffold) at day 21, whereas asterisk (*) shows that between different time points. No marking shows nonsignificance.

MC3T3-E1 cells were cultured on scaffolds constructed of 15% PCL with two different HA contents of 10% and 20% HA, respectively. Scaffolds were grouped according to their architecture into two groups, namely, uniform and FGS, and were cultured in mineralization medium. It was observed that the difference in architecture influenced viability (Figure 7A).

Cell viability of scaffolds showed a constant increase throughout the culture period. When uniform and FGSs were compared with 10% HA content, it was seen that the FGSs offered higher cell proliferation for up to 21 days. When HA content was increased to 20%, the advantageous effect of FGS was lost, and no significant difference in cell viability of uniform and FGS was observed. In fact, on the 21st day of culture, the cellular composition of all scaffolds was almost the same based on MTS data (Figure 7A).

#### 3.5.2 Assessment of ALP Activity

ALP activity was examined for the same uniform and FGS samples with two different HA % contents for which metabolic activity control was analyzed. There was an increasing ALP activity for all scaffolds throughout the 3 week culture period (Figure 7B). The results of the ALP enzyme activity showed that uniform structures had slightly higher ALP enzyme activity. For the ALP study, there was no statistically significant difference between the groups nor time points. One-way ANOVA was used for the statistical analysis, as stated in Section 2.9. Uniform scaffolds with 10% HA content showed higher ALP activity compared to that of FGSs. Based on the earlier observation that the percentage of cell viability was at its lowest in the HA10 uniform scaffolds within 21 days of culture, it can be argued that osteogenesis in this scaffold group is more prominent than proliferation. In other words, cells within HA10 uniform scaffolds demonstrate a greater inclination toward osteogenic differentiation rather than proliferation. On the other hand, increasing HA concentration in uniform scaffolds inversely affected ALP activity, where HA10 uniform scaffolds exhibited higher ALP activity than HA20 uniform scaffolds at all time points. On the contrary, increasing HA concentration resulted in increased osteogenic activity in FGSs, where HA20 FGS exhibited higher ALP activity than HA10 FGS.

#### 3.5.3 Cell Morphology Analysis of the 3D Scaffold under SEM

To obtain morphological information on how cells are attached to the 3D PCL-HA scaffolds with two different geometries and different HA contents, cells were imaged under FE-SEM on the 7th, 21st, and 28th days (Figure 7C). It was observed that on both uniform and FGSs with two different HA contents, PCL15HA10 and PCL15HA20, cells demonstrated considerable occupancy on the surface of scaffolds. On the 21st day, it was observed that the cells occupied the majority of scaffold strut surfaces, with some bridging over the pores between struts. On the 28th day, the struts were covered with an even more increased cell density. Overall, the scaffold surface, rather than the pore regions, was almost completely covered by interconnected cells in uniform scaffolds; however, in FGSs, there was evidence showing a connection between struts over the pores, particularly in the smaller pore size regions. More specifically, FGSs display extended filopodia and bridges between cells, whereas lamellipodial contact of cells is observed on the surface of uniform scaffolds. When compared to the 7th-day images, the existence of cell movement across macropores from struts can be argued. FGSs display more extended filopodia and multiple bridges between cells as compared to uniform samples on day 21 when compared to day 7. Similarly, lamellipodial contact of cells is pronounced on the surface of uniform scaffolds at day 21 when compared to day 7. On day 28, for uniform scaffolds, the scaffold surface, rather than the pore regions, is almost totally covered with connected cells, whereas in FGSs, there is a clear overarching between struts over the pores.

#### 3.5.4 Phalloidin Staining of 3D PCL/HA Scaffolds

**Figure 8:**
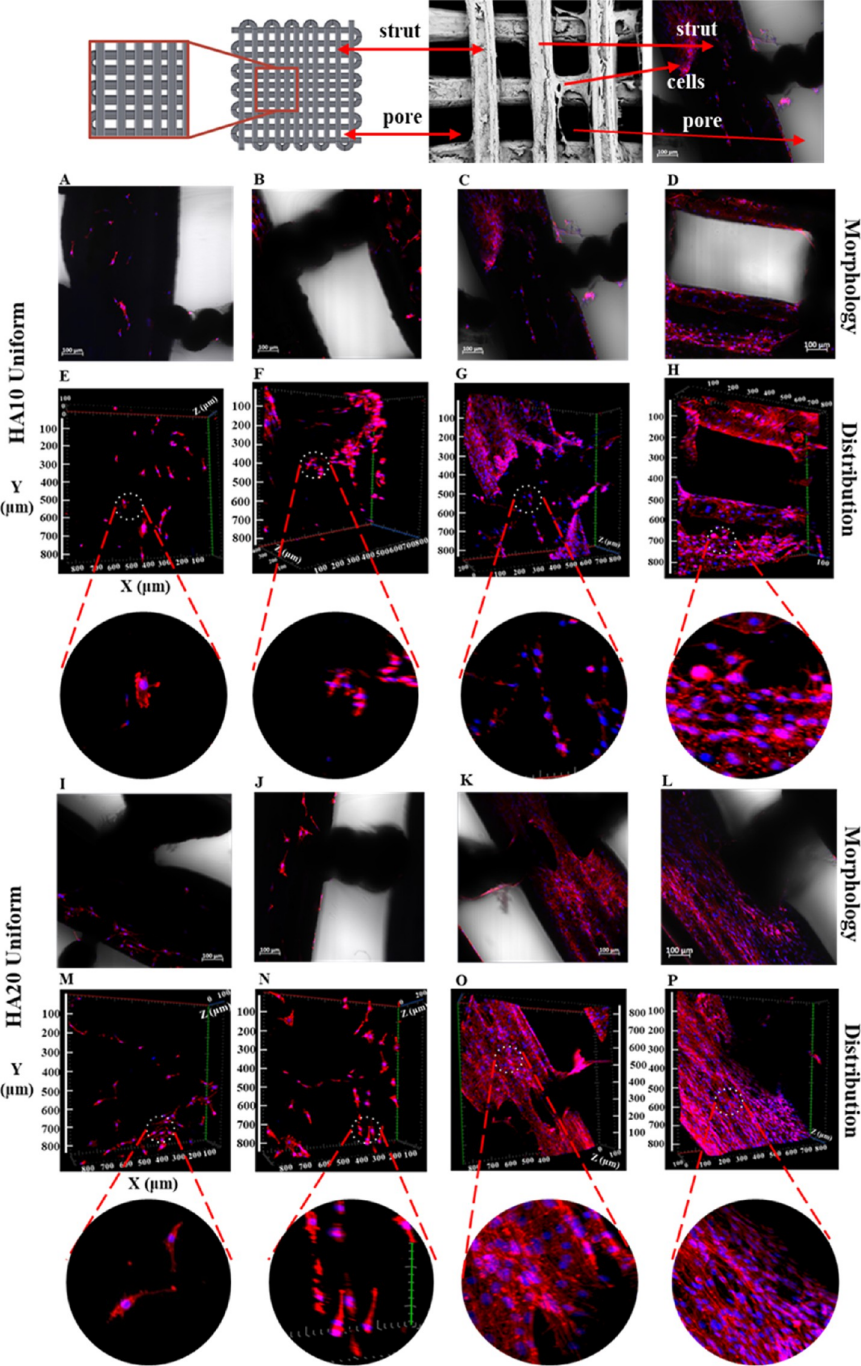
Morphologies (A–D,I–L) and distributions (E–H,M–P) of MC3T3-E1 cells cultured on uniform scaffolds, which have 10% HA (A–H) and 20% HA (I–P) for 7 (A, E, I, M), 14 (B, F, J, N), 21 (C, G, K, O), and 28 (D, H, L, P) days culture period by applying staining with phalloidin (red) and DAPI (blue).

**Figure 9:**
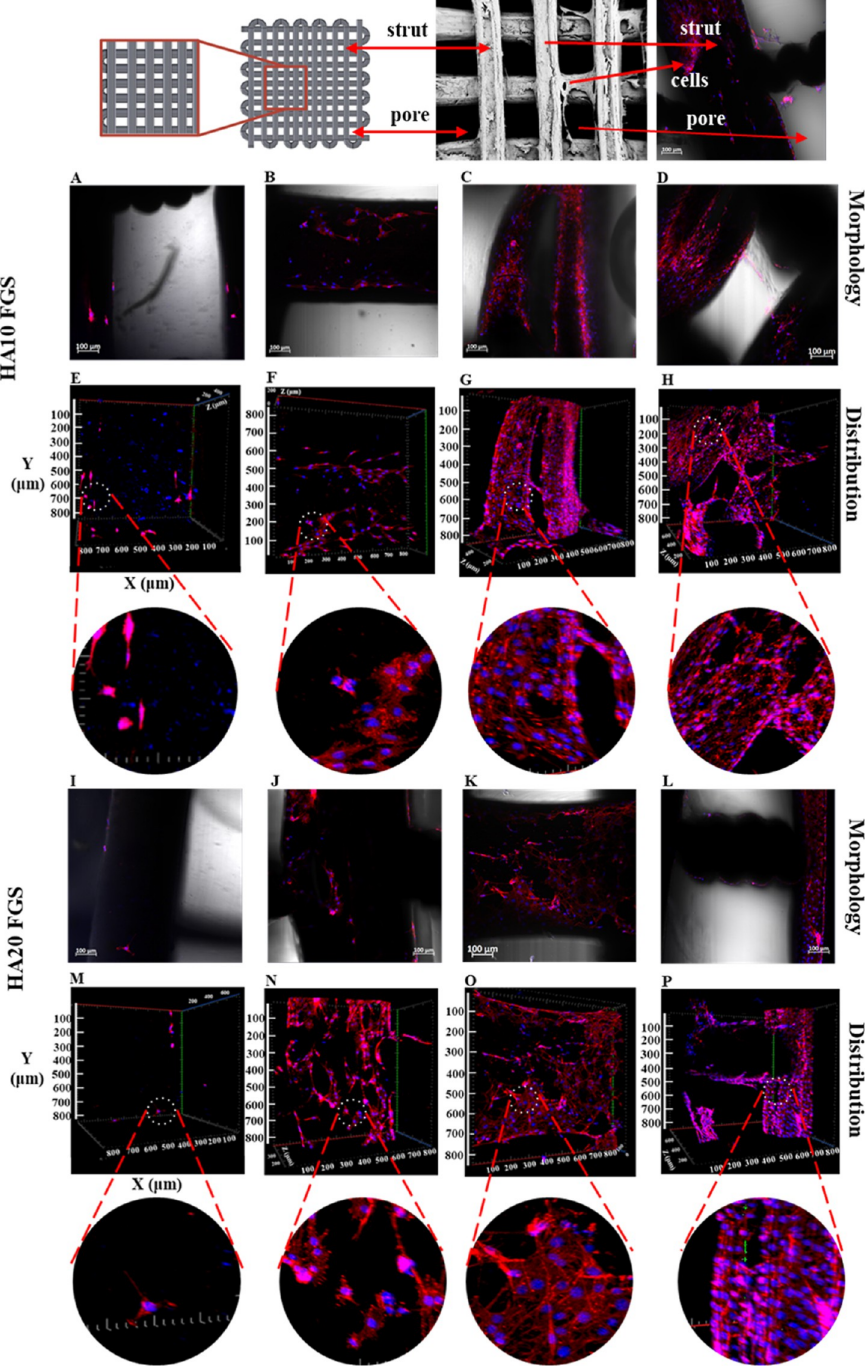
Morphologies (A–D,I–L) and distributions (E–H,M–P) of MC3T3-E1 cells cultured on FGSs, which have 10% HA (A–H) and 20% HA (I–P) for 7 (A, E, I, M), 14 (B, F, J, N), 21 (C, G, K, O), and 28 (D, H, L, P) days culture period by applying staining with phalloidin (red) and DAPI (blue).

To observe the cell distribution and morphology effect of uniform and FGSs containing 10% and 20% HA, a staining process was carried out at 7, 14, 21, and 28 days of culture (Figures 8 and 9). Cell adhesion and proliferation in scaffolds with both HA contents were detected based on the staining results. It was determined that each scaffold supports the growth of cells in the tissue scaffold (Figure 7). Cell proliferation and distribution were constantly increasing (based on the volume of cells, as seen in Figure 8E–H and M–P and Figure 9E–H and M–P) during the 28 day culture period on both functionally graded and uniform scaffolds. However, cell proliferation was favored on PCL15HA20 scaffolds with respect to PCL15HA10 scaffolds, especially on days 21 and 28 (Figure 8G, H and O, P), as also observed in the cell proliferation assay (Figure 7B). It is noted that phalloidin staining is used as a qualitative imaging analysis to confirm the MTS analysis. However, such imaging also provides visual observation of cell morphology and distribution. These experiments were repeated with 3 biological replicates, and pictures were taken from at least 5 different locations of the scaffolds. Keeping this in mind, the data provided (in a qualitative manner) are very consistent and in good agreement with MTS analysis, as previously stated.

FGSs exhibited cell attachment and proliferation constantly increasing during the 28 day culture period as well (Figure 8). The cell distribution on both HA contents was uniform, and the cell density was not different between 10% and 20% HA-containing scaffolds on days 21 and 28.

## 4 Discussion

In this study, PCL-HA composite scaffolds with two different morphologies, namely 3D uniform and FGSs, were fabricated by integrating 3D printing with NIPS. The FGS geometry includes a graded distance between the struts in the *x*- and *y*-axes, as well as in the *z*-direction. The objective was to construct a framework that closely mimics the diverse architecture, composition, and morphology observed in native bone tissue, as shown in Figure 1C. This was achieved with the inherent multiscale porosity, where the microporosity was induced via the NIPS process and macroporosity resulted from the as-designed and printed geometry shown in Figure 1B. Specifically, this work focused on several key considerations: (1) the feasibility of integrating 3D printing with the NIPS technique to produce uniform and FGS designs; (2) the relationship between design parameters (scaffold geometry, ceramic composition) on morphological, mechanical, and, more importantly, biological performance of scaffolds; and (3) the effect of incorporating gradient macroporosity while preserving microporosity within struts on these parameters.

The fabrication process demonstrated successful outcomes for PCL/HA scaffolds with 10 and 20 wt % HA contents, incorporating both uniform and FGS morphologies through the integration of the NIPS method with 3D printing. This approach delivered the desired simultaneous formation of micro and spatially controlled macropores within the scaffolds with overall porosities higher than 70% and a controlled macroporosity of approximately 61%, respectively, in all produced scaffolds matching desired porosity levels of actual bone^2^ as well as size ranges of macro- and micropore sizes for ideal scaffolds.

Before the parameters of 3D printing were optimized, the rheological properties of the prepared composite suspensions were assessed (Figure 2A–C). This involved examining the relationships between shear strain, shear stress, and viscosity. The objective was to anticipate the impact of polymer and ceramic concentrations on suspension viscosity and subsequently on 3D printing parameters such as printing pressure. Given the utilization of a pneumatic-based 3D printer in this study, equipped with a maximum 400 kPa supplier air compressor, it was also crucial to understand the machine’s limitations. Hence, the results of the rheology test and the calculated required air pressure (Figure 2C based on eq 1) indicated that the utilization of a 20% PCL suspension was impractical. Since this research involved the preparation of PCL suspensions using high molecular weight PCL pellets (80 kDa) dissolved in THF, any increase in its amount significantly raises the viscosity of the suspension due to the presence of long polymer chains. Suwanprateeb et al. demonstrated that the viscosity of the solution changes exponentially in relation to the concentration of the polymer.^35^ Our study’s findings were consistent with this study. Specifically, we observed that PCL20 exhibited a viscosity notably higher than that of PCL15. While the concentration of PCL had a more pronounced effect, increasing the HA content did not elevate the viscosity as much as the increase due to the PCL content. However, 20% HA resulted in a slight increase in viscosity and consequently a decrease in porosity and pore size, likely due to its influence on the rate of phase separation between THF and ethanol, making the demixing process faster and more instantaneous. This led to scaffolds with a higher HA content exhibiting less porosity and smaller pore sizes. The excessive concentration of polymer led to a viscosity that exceeded the specifications for printability by requiring a higher air pressure than the machine’s compressor could provide (Figure 2C). Hence, here, we explored the interplay among strain, stress, and viscosity. This relationship was established based on the Newtonian laminar flow assumption (eq 1), and the suspensions exhibited shear-thinning behavior, as demonstrated in Figures 2A,B.

Accordingly, the pressure and velocity values during printing were set according to the viscosity of the suspension. Given the nature of the NIPS method, the printed part of the scaffold was cured inside an ethanol bath during the printing process. Optimization of the velocity is critical, as printing too fast can lead to the collapse of successive vertical layers due to insufficient curing time. On the contrary, printing too slowly can result in the cured layers detaching from the glass surface of the ethanol bath, causing subsequent layers to deviate from the predetermined coordinates as dictated by the slicing software. In addition to setting a suitable velocity, the pressure used during printing is of great importance. High pressure levels can deposit excess material, building thicker struts that may merge, while low pressure may lead to insufficient material deposition, causing a lack of integrity between layers or early curing and detachment from the bottom of the ethanol bath. Successful optimization of velocity and pressure levels relative to each other ensures the production of well-printed uniform and FGSs.

As illustrated in Figure 3A,B,D scaffolds were consistently and accurately printed, showcasing a high fidelity to the original CAD design. This outcome reflects the reliability of extrusion (pneumatic)-based printing, a method demonstrated in recent studies.^35−37^ The morphological properties of the scaffolds, including microporosity and pore size, were assessed by using micro-CT tomography. It was observed that porosity and pore size decreased with increasing HA content, possibly due to an increase in viscosity and a change in phase separation rate. This behavior is also typically observed in producing composite scaffolds with HA addition using pore induction techniques such as solvent casting/salt-leaching.^42^ In the NIPS method employed here, the higher ceramic content may result in faster/instantaneous demixing between polymer rich and solvent rich phases. Additionally, SEM imaging was conducted to reveal surface porosity information for primarily a qualitative assessment of morphological surface features on both cross sections and the outer surface of 3D-printed uniforms and FGSs. For each geometry type, cross-sectional and surface images were captured on the same scaffold. Several areas on each scaffold were examined from both viewpoints, and a representative image that reflected the most common features was selected. Additionally, the same region was imaged at different magnifications to better assess the influence of that specific location on the scaffold’s morphology.

It is noted that the surfaces of the printed scaffolds exhibited minimal to no porosity across all groups. The discrepancy of views regarding surface porosity in PCL-HA composite films produced using NIPS exist. Sohn et al.^43^ claim in their study that the surface porosity only exists if the mold for casting of NIPS suspensions is covered, but surface pores do not exist in an uncovered mold in contrast to the study by Kim et al.^37^ Here, we attribute the absence of surface porosity to the high shear exerted by the nozzle during the 3D printing process. In addition, there is no significant difference in surface porosity between the groups based on SEM image analysis. In an earlier study, it was demonstrated that surface porosity is present, particularly on the bottom surface of the film scaffolds.^29^ Despite the lack of surface porosity, it could be argued that internal porosity, with its ability to change surface topography and roughness, could potentially lead to enhanced cell adhesion. A more detailed follow-up study would be needed to look into the precise inter-relationship between internal porosity and surface topography and its effect on cell adhesion. In our work, the cells adhered well to the scaffold surface and adopted an elongated morphology. Additionally, they extended across the macro channels, forming cellular bridges between scaffold struts.

The fabricated scaffolds were evaluated to ascertain the material decomposition and residual amount by performing TGA characterization, as shown in Figure 5. The quantity of HA residuals at the end of the TGA matched well with the experimental HA concentration. The residual amount in the 10% HA scaffold group closely matched the experimental value, whereas there was a slight deviation in the 20% group. This outcome could be attributed to potential agglomeration or loss in the 20% scaffold group. Furthermore, decomposition temperature decreased with increasing HA content aligning with the reported studies.^38,39^

To characterize the mechanical properties of the FGSs and uniform scaffolds, uniaxial compression and tension tests were performed. In alignment with findings in the literature,^31,37^ the compression and tension moduli exhibited an increase with higher ceramic content for both FGSs and uniform scaffolds. Additionally, introducing a gradient distance (macroscale) between filaments to FGSs slightly elevated the moduli of FGSs compared to their uniform scaffold counterparts. The alteration in mechanical properties has also been noted in studies of Sobral et al.^39^ and Bittner et al.,^40^ which emphasized the influence of design and porosity. The closer proximity of filaments, hence, local lower density, in the center region of the FGSs might have potentially improved structural integrity, despite equal porosity per design. It was hypothesized that an increased mechanical performance could be obtained by varying the pore distribution, allowing greater load-bearing capacity^12^ favorable for osteogenic differentiation, and HA was incorporated within the graded scaffolds to further assist with osteogenic differentiation, similar to the designs presented here but within scaffolds displaying controlled macropores. Also, based on the absorbed energy up until the fractured point, the toughness values indicate that FGSs display a higher ability to absorb energy than uniform scaffolds in contrast to their tensile modulus values. This aligns well with results focusing on FGS designs.^19^

To be able to evaluate the biological performance of 3D-printed structures, cell viability and ALP enzyme activity were conducted with MC3T3-E1 cells. Throughout the cell culturing period in this study, the cell viability of the scaffolds consistently increased, regardless of design and HA composition. In comparison between uniform and FGSs with 10% HA content, the FGSs demonstrated a higher cell proliferation, up to 21 days. This outcome may be attributable to the radially changing gradient distance between filaments in the center of FGSs, potentially leading to higher seeding efficiency, which was also observed by Sobral et al.^39^ as well as Nowicki et al.^12^ More specifically, in a previous work,^12^ 3D printing of osteochondral scaffolds with graded microstructure via FDM resulted in improved cell adhesion and proliferation in the graded pore structures when compared to homogeneously distributed porous and nonporous structures. However, based on our results, with an increase in HA content to 20%, the advantageous effect of the FGS waned, and no significant difference in cell viability between uniform and FGSs was observed. By the 21st day of the culture, the cellular composition of all scaffolds was nearly identical based on MTS data. These findings align with those of previous studies, highlighting a correlation between pore size and its distribution and the scaffolds’ ability to support cell growth and proliferation, a key theme in FGSs research.^19,21^ This line of research has notably investigated pore gradients (macroscale) within FGSs, revealing enhancements in cell proliferation rates, as well as improved ALP activity and the upregulation of osteocalcin and osteopontin regulations.^22,23^ However, a potential discrepancy between our findings and those in existing literature is that uniform scaffolds containing 10% HA exhibited ALP activity that was higher than that of FGSs. The lower cell viability percentage observed in the HA10 uniform scaffolds over a 21 day culture period implies a stronger effect on osteogenesis than on proliferation within this specific scaffold group. In simpler terms, cells within the uniform HA10 scaffolds tend to experience osteogenic differentiation instead of proliferation. Conversely, an increase in the HA concentration in uniform scaffolds had a contrasting effect on ALP activity, with HA10 uniform scaffolds showing higher ALP activity than their HA20 counterparts at all time points. On the other hand, an increase in HA concentration resulted in enhanced osteogenic activity in FGSs, evident from the higher ALP activity in HA20 FGS than in HA10 FGS. However, in the current ALP study, neither a statistically significant difference between the groups nor time points was observed.

In conclusion, a comprehensive analysis of structural, compositional, and biological characteristics of the scaffolds indicated that both uniform and FGSs with multiscale porosity offered a suitable environment for bone tissue regeneration. Moreover, FGSs closely mimicking the macrogradient pore morphology of native bone with high overall porosity and designed pore gradients have resulted in improved mechanical and biological responses, suggesting that FGS morphology with dual porosity is likely to provide a more favorable environment for cellular response when compared with uniform dual porosity scaffolds.

## 5 Conclusions

Bone tissue’s intricate hierarchical structure undergoes continuous remodeling, necessitating effective scaffolds for regeneration. Numerous studies have been conducted on the complicated structural and morphological changes observed in natural bone, mainly by designing FGSs that display gradients in macropore size between struts. This study investigates the significance of multiscale pores, where micro- and gradient macroporosity coexist, addressing a gap in current literature that primarily focuses on either gradient macrosized pores or uniform multiscale porosity. Towards that goal, we successfully integrated 3D printing with the NIPS technique to fabricate 3D PCL/HA composite scaffolds with uniform and functionally graded structures. Characterization revealed that these scaffolds exhibited distinct internal micropore and macropore morphologies, providing adequate strength and supporting cell proliferation, as confirmed by mechanical analysis and biological assessments. Our results indicated that increasing the HA content enhanced scaffold strength but reduced porosity and pore size. Notably, FGSs with gradient distances between filaments demonstrated improved structural integrity. Biological assessments showed that while uniform scaffolds with 10% HA promoted osteogenesis, FGSs with 20% HA facilitated both proliferation and osteogenesis of preosteoblast cells. In conclusion, FGSs with multiscale porosity offer strong potential for bone tissue regeneration. Future work should optimize material compositions, examine the effect of HA distribution, explore cellular behavior, and pursue patient-specific designs to enhance the application of scaffolds in bone tissue engineering.

## Supporting Information Available

Experimental procedure for cell culturing, including ALP activity assessment for optimum reaction time and mineralization medium evaluation and Alizarin Red S staining for mineralization medium evaluation (PDF)
