# Supplementary material for: 3D-Printed Functionally Graded PCL-HA Scaffolds with Multi-Scale Porosity
Source: ACS Omega. 2025 Feb 14;10(7):6502–19. doi: 10.1021/acsomega.4c06820 (PMC11866177; doi:10.1021/acsomega.4c06820)
Supplement: Supplementary file 1 — ao4c06820_si_001.pdf [file ao4c06820_si_001.pdf]

3D Printed Functionally Graded PCL-HA Scaffolds with Multi-Scale Porosity

Supporting Information

Hatice Kubra Bilgili[a,b], *Mehmet Serhat Aydin*[a,c], *Mervenaz Sahin*[b], *Sevilay Sahin*[d], *Sibel Cetinel*[f], *Gullu Kiziltas*[e,f*]

a Department of Material Science and Nanoengineering, Faculty of Engineering and Natural Sciences, Sabanci University, Istanbul, Turkey

b Division of Human Mechanical Systems and Design, Graduate School of Engineering, Hokkaido University, Sapporo, Japan

c Center for Translational Oral Research (TOR), Department of Clinical Dentistry, Faculty of Medicine, University of Bergen, Norway

d Department of Molecular Biology, Genetics and Bioengineering, Faculty of Engineering and Natural Sciences, Sabancı University, Istanbul, Turkey

e Department of Mechatronics, Faculty of Engineering and Natural Sciences, Sabanci University, Istanbul, Turkey

f Sabanci University Nanotechnology Research and Application Center, Istanbul, Turkey

*Corresponding Author: Prof. Dr. Gullu Kiziltas

E-mail: gkiziltas@sabanciuniv.edu

Characterization of HA Powder

Commercially available HA powder (<200 nm; Sigma Aldrich, St. Louis, MO, USA; Lot number: MKCM1889) was used to prepare the suspension. Characterization studies using SEM, EDX and XRD analysis were carried out using the HA powder. Resulting plots of EDX and XRD show similarity with available data in literature (Manafi et al., 2008).

1. **Scanning Electron Microscopy (SEM) analysis results of HA powder used for the preparation of suspensions**

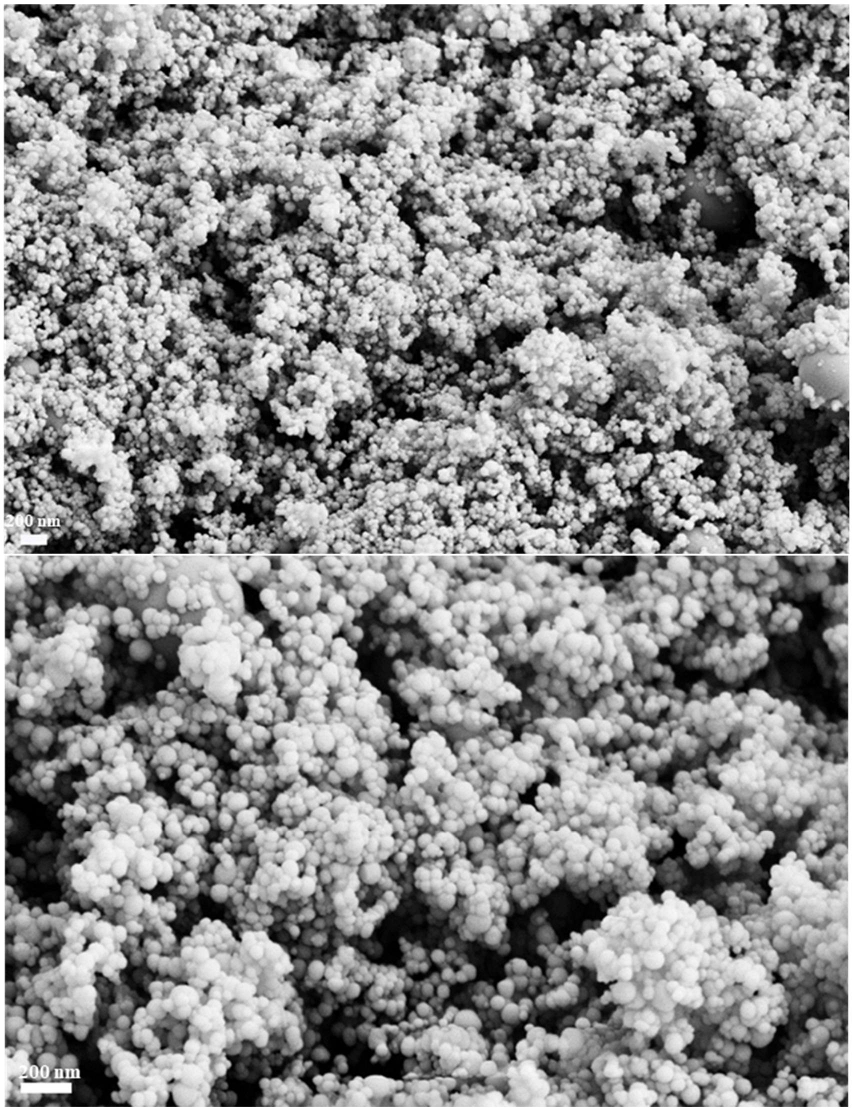

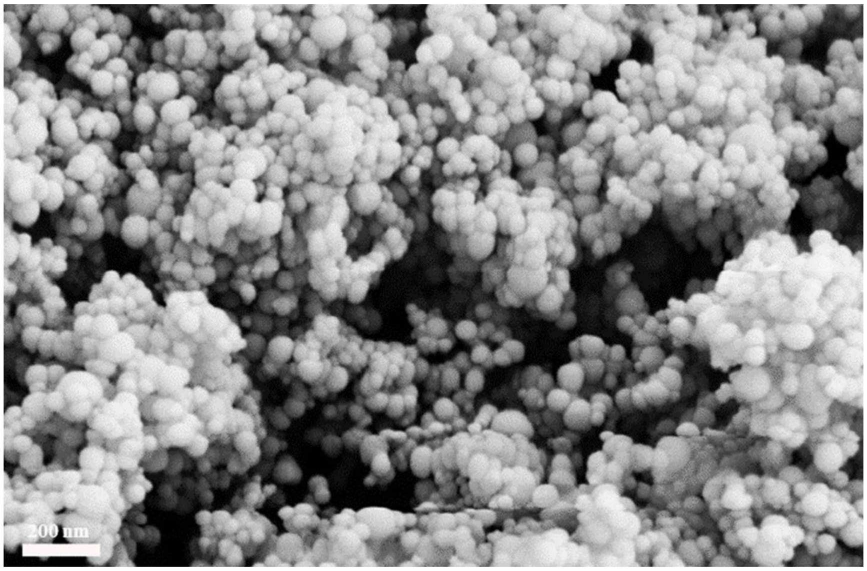

Figure S1 SEM analysis of HA powder under different magnifications from top to bottom magnifications are: 50k, 100k,150k, respectively.

2. Energy dispersive X-ray (EDX) Analysis

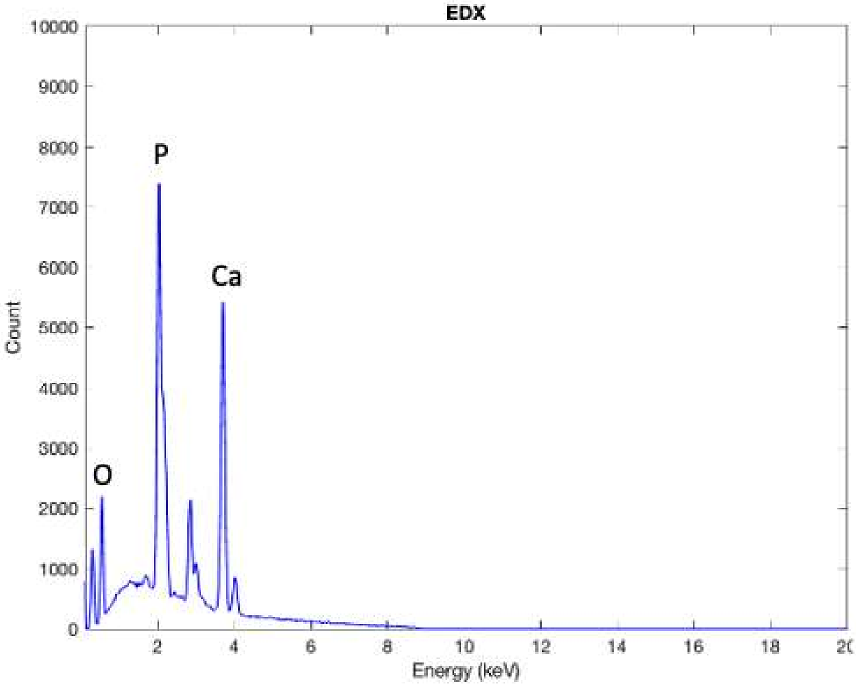

Figure S2 Energy dispersive x-ray analysis (EDX) of the HA powder used for the preparation of the suspension.

The EDX pattern showed expected characteristic peaks of O, P and Ca as cited in literature (Manafi et al., 2008).

Manafi, S. A., Yazdani, B., Rahimiopour, M. R., Sadrnezhaad, S. K., Amin, M. H., & Razavi, M. (2008). Synthesis of nano-hydroxyapatite under a sonochemical/hydrothermal condition. *Biomedical Materials, 3*(2), 025002. https://doi.org/10.1088/1748-6041/3/2/025002

3. X-Ray Diffraction (XRD) Analysis

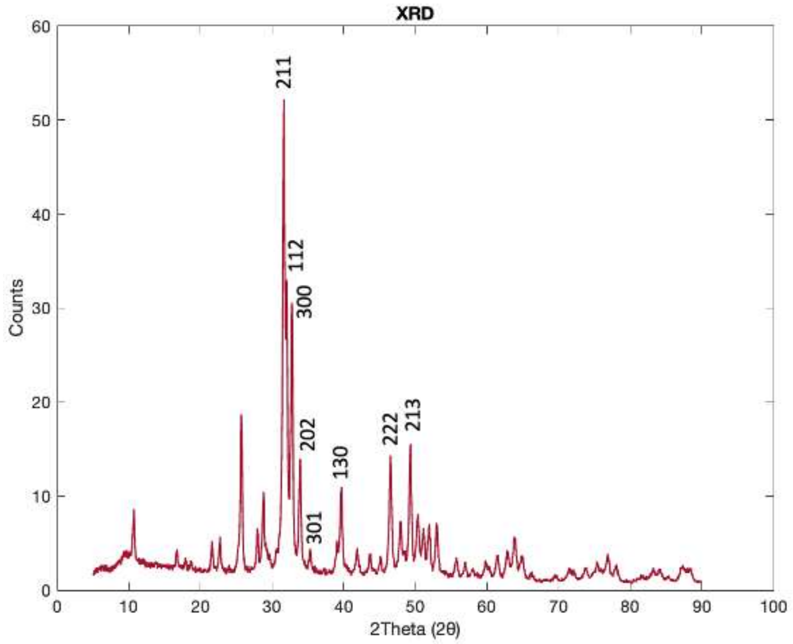

Figure S3 X-Ray Diffraction (XRD) analysis of the hydroxyapatite.

HA powder was analyzed by X-ray diffraction (XRD) technique by using D2 PHASER Desktop diffractometer (Bruker, Billerica, MA, USA) employing CuKα radiation. X-ray diffraction patterns were recorded in the angular range 2θ = 5–90◦. The XRD patterns in Figure S1.3 show very similar peaks as cited in literature (Chandrasekar et al., 2013) and the corresponding peaks were determined and noted on the plot.

Chandrasekar, A., Sagadevan, S., & Dakshnamoorthy, A. (2013). Synthesis and characterization of nano-hydroxyapatite (n-HAP) using the wet chemical technique. *International Journal of Physical Sciences, 8*(32), 1639-1645. https://doi.org/10.5897/IJPS2013.3990

Mechanical Characterization

The ultimate tensile stress and compression stress were provided in the below table with the corresponding values for two types of scaffolds and different HA percentages.

Design (From stress strain curve)	HA (%)	max tension stress (MPa)	max tension strain (mm/mm)	max comp stress (MPa)	max comp strain (mm/mm)
Uniform	HA10	0.6 ± 0.05	1.54 ± 0.12	4.5 ± 1.19	1.32 ± 0.05
	HA20	0.9 ± 0.06	1.03 ± 0.34	5 ± 1.45	1.22 ± 0.1
FGS	HA10	0.83 ± 0.08	2.38 ± 0.48	8.47 ± 0.34	1.6 ± 0.25
	HA20	1.08 ± 0.1	2.34 ± 0.72	8.67 ± 0.28	1.35 ±

					0.06

Table S1 The ultimate tensile stress and compression stress were provided in the below table with the corresponding values for two types of scaffolds and different HA percentages.

Ultimate/maximum tensile strength from the stress-strain curve was found as 0.6 ± 0.05, 0.9 ± 0.06, 0.83 ± 0.08, 1.08 ± 0.1 MPa for HA10Uniform, HA20Uniform, HA10FGS and HA20FGS groups, respectively. Similarly, maximum compressive strength from the stress-strain curve was found as 4.5 ± 1.19, 5 ± 1.45, 8.47 ± 0.34, 8.67 ± 0.28 MPa for HA10Uniform, HA20Uniform, HA10FGS and HA20FGS groups, respectively. Whereas maximum tensile strain was found as 1.54 ± 0.12, 1.03 ± 0.34, 2.38 ± 0.48, 2.34 ± 0.72 mm/mm for HA10Uniform, HA20Uniform, HA10FGS and HA20FGS groups, respectively. Similarly, maximum compressive strain was found as 1.32 ± 0.05, 1.22 ± 0.1, 1.6 ± 0.25, 1.35 ± 0.06 mm/mm for HA10Uniform, HA20Uniform, HA10FGS and HA20FGS groups, respectively.

Cell Culturing and Mineralization Medium

Cell culture studies were carried out by MC3T3-E1 Subclone 4 (ATCC CRL-2593). Alpha-MEM (Gibco) supplemented with 10 % Fetal Bovine Serum (Pan Biotech) and 1% Penicillin-Streptomycin solution (Pan Biotech) was used as a growth medium.

Two types of culture media were tried to select the most suitable condition for mineralization. The first of these (Mineralization Medium-1) was obtained by adding 10µg/mL ascorbic acid (Sigma) and 3mM β-Glycerophosphate (Sigma) to the growth medium, while the other (Mineralization Medium-2) was obtained by adding 50µg/mL ascorbic acid and 10mM β-Glycerophosphate to the growth medium.

Assessment of Alkaline Phosphatase Activity

Since the alkaline phosphatase (ALP) enzyme is an extracellular enzyme, a sample taken from the culture medium was used for the experiments. ALP Detection Kit (Sigma) was used where 2 µL p-nitrophenyl phosphate (pNPP) and 2 µL culture medium were added to 96 µL of reaction solution. At 405 nm, absorbance measurements were taken. In order to find the optimum reaction time, measurements were taken at 15-minute intervals for 1 hour. Then, in the study carried out after 7, 14, 21 and 28 days of culture, the ALP activity of the cells was observed during this period. Based on these measurements, an average of two values were obtained for samples collected from each medium, and the findings were assessed.

In order to determine the optimum reaction time, the measurement results taken for 1 hour at 15-minute intervals are given in Figure S4. It was concluded that the optimal reaction time for the ALP assay was 45 minutes.

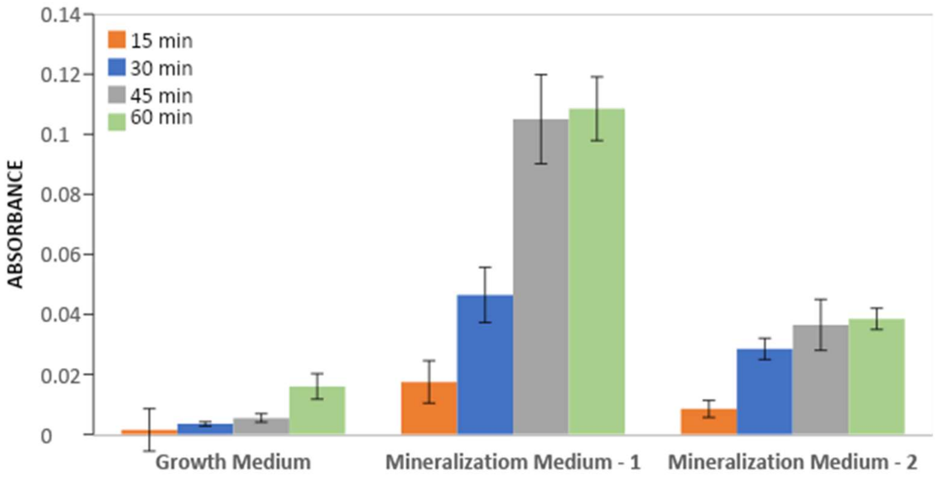

Figure S4 Determination of the optimum reaction time for the ALP assay.

Measurements were made every week for 28 days of culture and show that the most suitable mineralization medium was Mineralization Medium-1, which showed the highest ALP activity in the first 7 days (Figure S5).

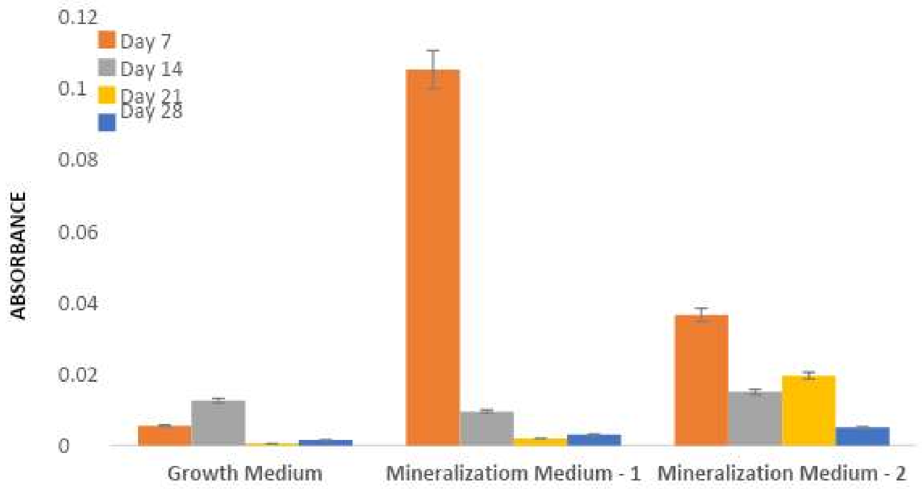

Figure S5 ALP enzyme activities in 4 different culture periods of MC3T3-E1 Subclone 4 cells cultured in two different mineralization media and growth media.

Based on this data, ALP activity was highest in the first week of the Mineralization Medium-1 group, but it gradually decreased over time. The observed decrease in activity was concluded to be owing to a lack of suitable living space due to the excessive increase in cell density in the later time, and this observation was taken into account in later experiments.

Alizarin Red S Staining for Medium Evaluation

Alizarin Red S staining was performed in order to evaluate the calcium deposition formed by mineralization. 2% Alizarin Red S (Sigma) was dissolved in sterile ddH_2O, and its pH was adjusted to

4.1-4.3. After the culture medium was collected and the samples were washed with sterile PBS, Alizarin Red S dye solution was added to cover the samples completely and incubated at room temperature for 15 minutes. After the dye was collected, the samples were washed with sterile PBS until all dye residues were removed and images were taken under an inverted microscope (Zeiss, Provert).

Alizarin Red S staining results of the samples cultured in control medium and two different mineralization media for 7, 14, 21 and 28 days are shown in Figure S6. The result shows that the greatest calcium accumulation was obtained by culturing in Mineralization medium-1.

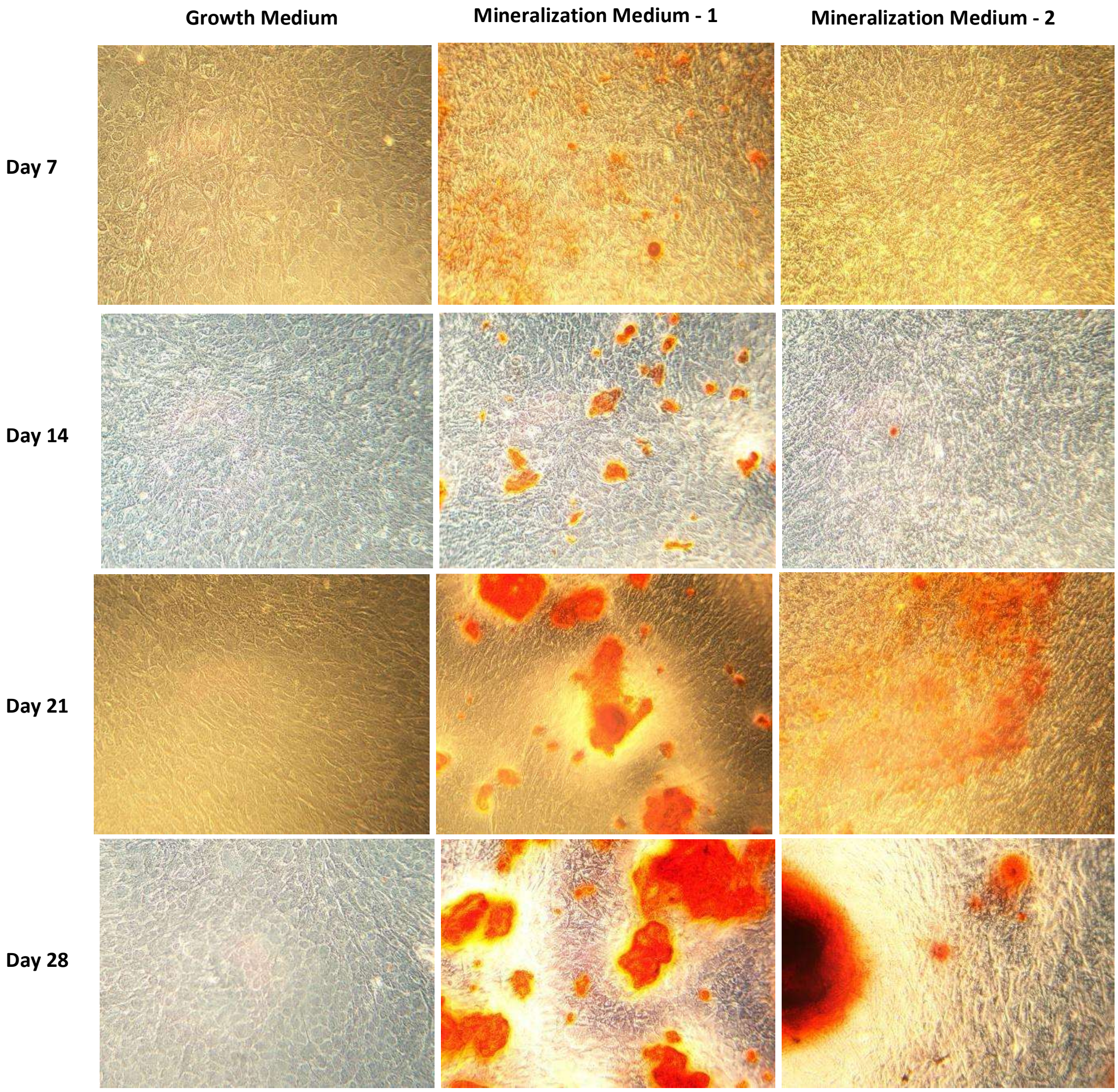

Figure S6 Alizarin Red S staining result obtained by culturing MC3T3-E1 Subclone 4 cells in growth medium and two different mineralization mediums (10X Magnification).

Mineralization Medium-1 was chosen for mineralization experiments to be carried out on scaffolds as a consequence of ALP and Alizarin Red S staining.

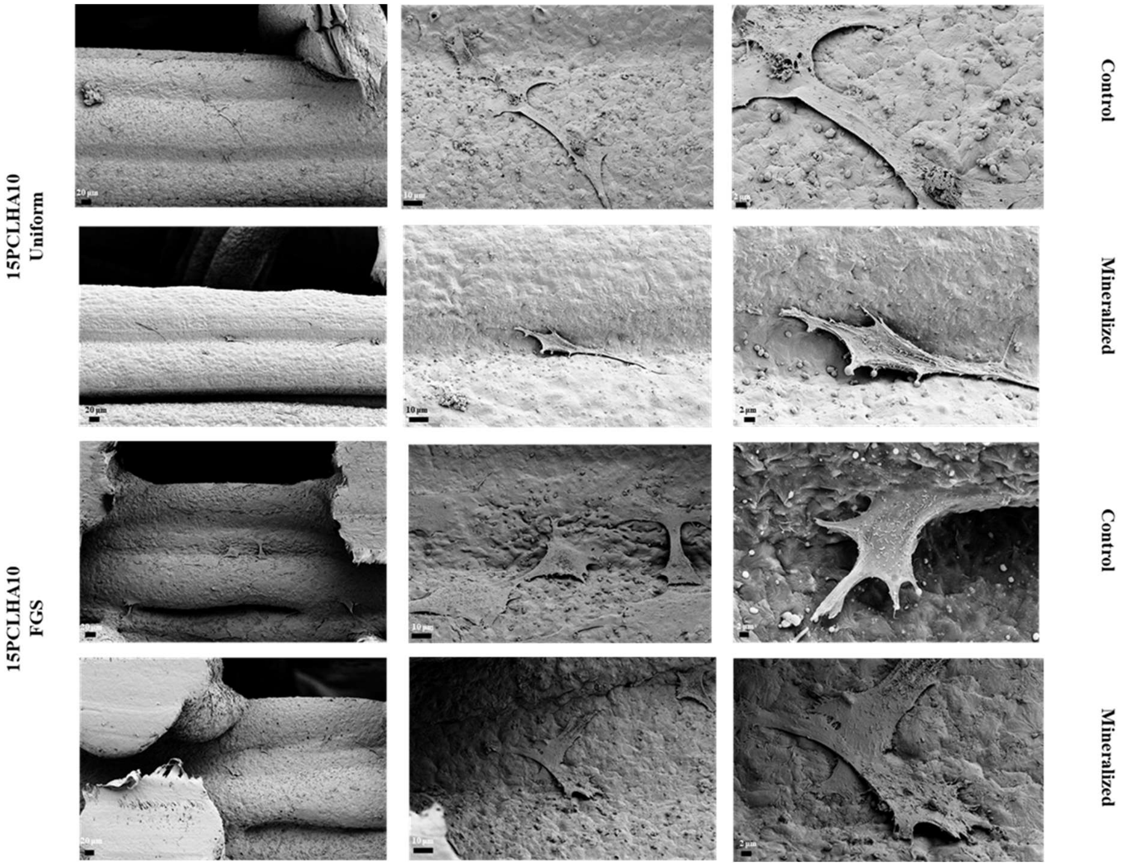

Figure S7 SEM images of inner layers of scaffolds with cells of uniform and FGSs with 10% HA content (7th day).
